# Expression and localization of two β-carbonic anhydrases in *Bienertia*, a single-cell C_4_ plant

**DOI:** 10.3389/fpls.2024.1506375

**Published:** 2025-01-16

**Authors:** Tho Nguyen, Nakyoung Lee, Fabian J. Frömling, Toni L. Meister, Jung Sun Kim, Sascha Offermann, Inhwan Hwang

**Affiliations:** ^1^ Department of Life Sciences, Pohang University of Science and Technology, Pohang, Republic of Korea; ^2^ Clinic for Hematology, Oncology, Infectiology and Palliative Medicine, The Karlsruhe Municipal Hospital, Karlsruhe, Germany; ^3^ Institute for Infection Research and Vaccine Development (IIRVD), Centre for Internal Medicine, University Medical Centre Hamburg-Eppendorf (UKE), Hamburg, Germany; ^4^ Department for Clinical Immunology of Infectious Diseases, Bernhard Nocht Institute for Tropical Medicine (BNITM), Hamburg, Germany; ^5^ German Centre for Infection Research (DZIF), Partner site Hamburg-Lübeck-Borstel-Riems, Hamburg, Germany; ^6^ Department of Agricultural Biotechnology, National Institute of Agricultural Sciences, Rural Development Administration, Jeonju, Republic of Korea; ^7^ Institute for Botany, Leibniz University Hannover, Hannover, Germany

**Keywords:** β-carbonic anhydrases, *Bienertia*, cytosol, plasma membrane, palmitoylation

## Abstract

Carbonic anhydrases (CAs) are ubiquitous enzymes that catalyze reversibly both the hydration and dehydration reactions of CO_2_ and HCO_3_-, respectively. Higher plants contain many different isoforms of CAs that can be classified into α-, β- and γ-type subfamilies. β-type CAs play a key role in the CO_2_-concentrating mechanism, thereby contributing to efficient photosynthesis in the C_4_ plants in addition to many other biochemical reactions in plant metabolism. Here, we characterized at the molecular, cellular and biochemical levels two β-type CAs in *Bienertia sinuspersici*, a plant that operates a C_4_ carbon concentrating mechanism within individual cells without the Kranz anatomy. These two β-type CAs (BsCAβs), named BsCAβ1 and BsCAβ2, in *Bienertia* were strongly induced along with maturation of leaves. Both BsCAβ1 and BsCAβ2 existed as a dimeric form *in vivo* but showed differential localization. BsCAβ2 was localized exclusively to the plasma membrane in *Bienertia* and when expressed heterologously in the C_3_ Arabidopsis. In contrast, BsCAβ1 largely localized to the cytosol together with a portion to the plasma membrane (PM) in both plants. BsCAβ2 had two cysteine residues at the N-terminal region for palmitoylation and their substitution with serine residues led to a change in the localization from the plasma membrane (PM) to the cytosol. Thus, we propose that BsCAβ2 localizes to the PM using a lipid moiety added posttranslationally plays a role in conversion of cytosolic CO_2_ into HCO_3_- as part of the CO_2_-concentrating mechanism, thereby contributing to the single-cell C_4_ photosynthesis in *Bienertia*.

## Introduction

Photosynthetic organisms fix CO_2_ from the atmosphere into glucose in the chloroplast, to use as an energy source and the starting material for production of various cellular compounds. Therefore, the efficiency of CO_2_ capturing is closely related to the photosynthetic efficiency, and the yield of plants (Kant et al., 2012). Most plants including major crops such as rice and wheat belong to the C_3_ photosynthetic type, named after the primary fixation product namely 3-phophoglycerate (a three carbon containing organic acid) (Häusler et al., 2002). In the C_3_ plants, atmospheric CO_2_ enters through the stomates and diffuses directly into chloroplasts where it is fixed into sugars (Rawsthorne, 1992; Medrano et al., 2002; Pego et al., 2000). Thus, the CO_2_ level within mesophyll cells in the leaf tissue where photosynthesis takes place is critical for efficient photosynthesis. Certain plants have developed a CO_2_-concentrating mechanism (CCM) to increase cellular CO_2_ levels (Furbank, 2017; Leegood, 2002). In this process, CO_2_ is first converted to HCO_3_- by carbonic anhydrases in the mesophyll cells. Subsequently, HCO_3_- is conjugated to phosphoenolpyruvate (PEP) resulting in the production of C_4_ acids by PEP carboxylase (PEPC). Hence, this type of photosynthesis is termed the C_4_ photosynthesis. The C_4_ compounds produced in mesophyll cells diffuse into the neighboring bundle-sheath cells where CO_2_ is released again and is ultimately fixed into sugars by Rubisco and the enzymes of the reductive pentose-phosphate cycle. Through this mechanism, the C_4_ plants can enhance the photosynthetic efficiency by increasing CO_2_ levels at the Rubisco complex. In the CCM of the C_4_ plants, PEPC utilizes HCO_3_- rather than CO_2_ to produce C_4_ acids. Thus, one of the most critical steps is the rapid conversion of CO_2_ from atmosphere to HCO_3_- by CAs (Hatch and Burnell, 1990), emphasizing their crucial role for the CCM in the C_4_ plants.

Plant CAs are classified based on structural differences into three subfamilies; αCAs, βCAs and γCAs (Moroney et al., 2001; Hewett-Emmett and Tashian, 1996). In addition, recently two new families, named δ and ζCAs, were found that are largely present in algae (McGinn and Morel, 2008; Park et al., 2007). Plants have a large number of genes encoding CAs (DiMario et al., 2017). They are expressed in various tissues and show different subcellular localization such as chloroplasts, mitochondria and cytosol depending on their physiological roles (DiMario et al., 2017). The C_3_ plants have major CA activity in mesophyll cell chloroplasts (Gutierrez et al., 1974). In contrast, βCAs are largely responsible for HCO_3_- production in the cytosol of mesophyll cells in the C_4_ plants. A spatial separation of the sites of HCO_3_- production and CO_2_ consumption is required for high photosynthetic efficiency. Typically this is achieved by separation of HCO_3_- production within the mesophyll cells and separation of the final CO_2_ fixation reactions within the bundle sheaths cells of the C_4_ plants (Leegood, 2002).

Interestingly, several plant species were identified that carry out the C_4_ photosynthesis within individual photosynthetic cells and therefore without the need for separating primary and final carbon fixation reactions between two different cell types. The best studied example is *Bienertia sinuspersici* (Voznesenskaya et al., 2001, 2002) which operates a CCM within individual photosynthetic cells by using two dimorphic chloroplasts that are intracellularly partitioned into two different compartments (Edwards et al., 2004; Akhani et al., 2005). Chlorenchyma cells have a large central compartment that is packed with central chloroplasts and mitochondria and a peripheral compartment that contains peripheral chloroplasts (Akhani et al., 2005; Voznesenskaya et al., 2002). Each compartment is interconnected by cytoplasmic channels (Lara et al., 2008). Biochemical and physiological analysis indicates that the two cellular compartments in Bienertia basically mimic the function of mesophyll and bundle sheath cells of traditional Kranz type C_4_ species (Offermann et al., 2011).

In this study, we focused on CAs as an approach to get insight into the single cell C_4_ system in Bienertia. We provide evidence that two isoforms of β-type CAs, *BsCAβ1* and *BsCAβ2*, show gradual increases in the expression along with the maturation of leaf cells, and localize differentially to the cytosol and plasma membrane, respectively, as dimers. Moreover, posttranslational modification of BsCAβ2 by palmitoylation is necessary for the plasma membrane localization.

## Materials and methods

### Plant materials and growth conditions

Bienertia (*Bienertia sinuspersici*) plants were propagated by vegetative cutting asexually (Lung et al., 2011) and grown in an artificial growth chamber under a 16 h/8 h light/dark cycle at 25°C to 30°C. Approximately 2 weeks later, rooted plants were transferred to pots containing soil in the greenhouse. Plants were watered with 30 mM NaCl and nutritional supplement (BIO-NEX). Three month-old plants were used for experiments.

Arabidopsis (*Arabidopsis thaliana*, Col-0) plants were grown on B5 agar plates (3.16 g/L Gamborg B5 medium, 2% w/v sucrose, 2 mM MES (pH 5.7), 0.85% Agar) in an artificial growth chamber under a 16 h/8 h light/dark cycle at 20°C to 22°C. Leaves from 14 day-old plants were used for protoplast isolation.

### RNA sequencing

Total RNA was extracted from leaf tissue using an RNeasy mini prep kit (Qiagen, Hilden, Germany) and treated with DNase I (Qiagen) to remove residual DNA. Subsequently, cDNA libraries were prepared with a TruSeq stranded-mRNA Prep Kit (Illumina, CA, USA) as follows: mRNA molecules were purified from the total RNA using polyT beads and fragmented to an average length of 200−300 bp by Mg^2+^ catalyzed hydrolysis. Single-stranded cDNAs were generated from the mRNA fragments via random hexamer priming. Then double-stranded cDNA was prepared with second strand synthesis. The cDNA libraries were PCR-amplified after end-repair process, size selection (> 100 bp), A-tailing, and adapter ligation. Sequencing was progressed by the Illumina HiSeq 2500 machine (Illumina) according to the manufacturer’s protocols. Low quality reads were filtered at Q30 score, and the remaining reads (89%) were mapped by the Trinity method of *De novo* transcriptome assembly (Grabherr et al., 2011). Putative CA sequences of Bienertia are searched using BLASTX (Altschul et al., 1990), and insignificant matches are excluded (> 10^-10^ E-value, < 80% query coverage).

### Phylogenetic analysis

To confirm α and β type CAs, we compared them to paralogs of representative plants of monocot (*Oryza sativa* subsp. *japonica* and *Zea mays*) and dicot (*Arabidopsis thaliana*, *Spinacia oleracea*, and *Nicotiana tabacum*). Multiple sequences of CAs were aligned by MUSCLE with default parameter (Edgar, 2004), and the neighbor-joining (NJ) trees of both CAs were constructed with Mega X software using 1000 bootstrap replications, Poisson correction model, and pairwise deletion (**Supplementary Figure S1**; Zuckerkandl and Pauling, 1965; Kumar et al., 2018).

### Plasmid construction

*BsCAβ1* and *BsCAβ2* cDNAs were isolated by polymerase chain reaction (PCR) from cDNA library prepared from total RNA of Bienertia leaves using gene-specific primers that were designed based on the sequence information obtained from RNA seq (**Supplementary Table S1**). To generate the *BsCAβ2[C13,14S]* mutant construct, cysteine residues in position 13 and 14 were substituted to serines by PCR-based site-directed mutagenesis using specific primers (**Supplementary Table S1**), and *BsCAβ2* cDNA as template (**Supplementary Table S1**). For the expression in Arabidopsis protoplasts, PCR products were digested with Xba1/Xma1 restriction endonucleases and inserted into an GFP-containing expression vector under the cauliflower mosaic virus (CaMV) 35S promoter. For the expression in Bienertia protoplasts, PCR products were digested with XhoI/XmaI and inserted into a 35S:puc18-spGFP6 expression vector as described in Wimmer et al. (2017). All primers are listed in **Supplementary Table S1**.

**Figure 1:**
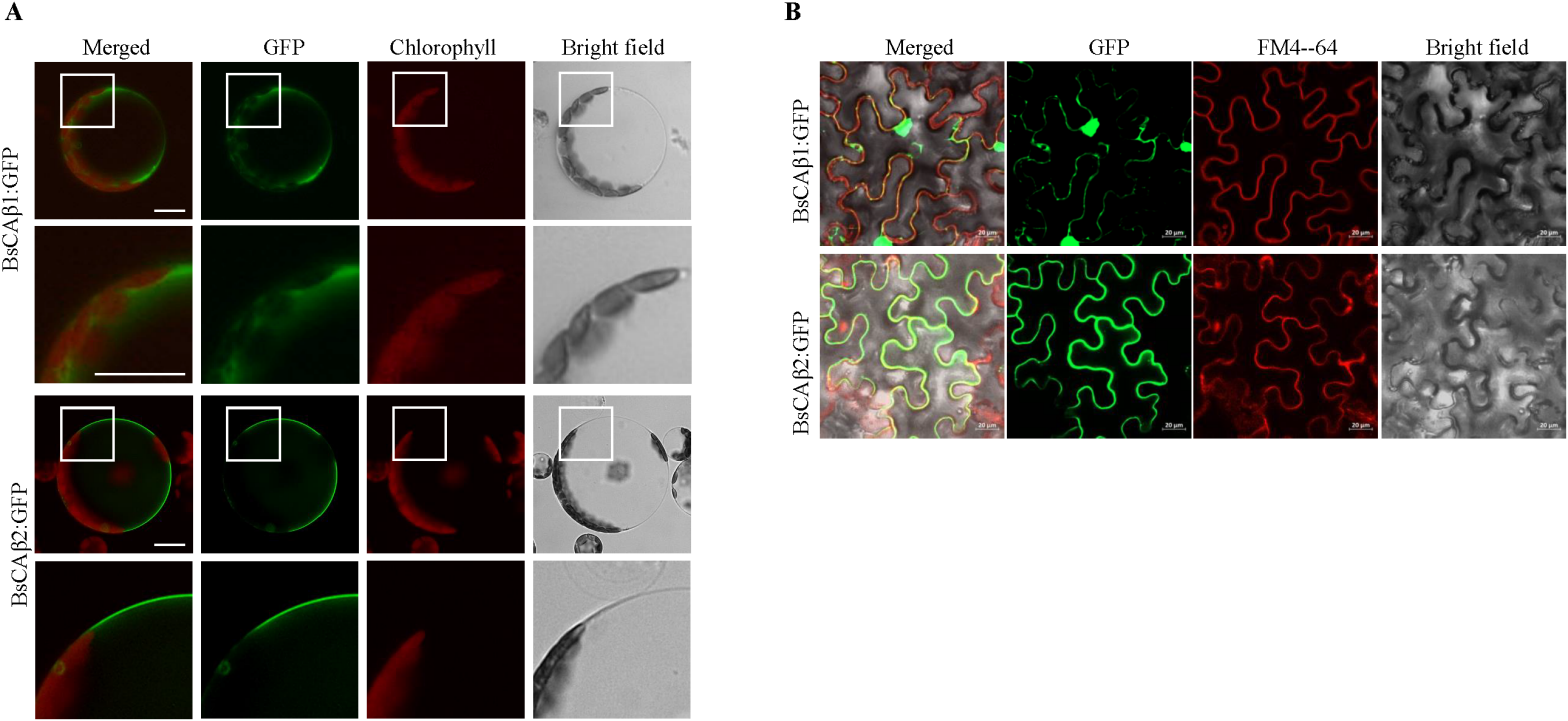
Subcellular localization of BsCAβs in Arabidopsis protoplasts and N. benthamiana leaves. **(A)** *In vivo* localization of BsCAβs:GFP in Arabidopsis protoplasts. Protoplasts were transformed with BsCAβs:GFP and the localization was examined 24 h after PEG transformation. GFP and autofluorescence of chlorophyll are shown in the green and red channels, respectively. Scale bar = 20 µm. **(B)** *In vivo* localization of BsCAβs:GFP in N. benthamiana leaves. BsCAβs:GFP were transformed into leaf cells of N. benthamiana via Agrobacterium-mediated infiltration. The localization of BsCAβs:GFP was examined 3 days of post-infiltration. GFP and FM4-64 dye are shown in the green and red channels, respectively. Scale bars = 20 µm, 30 cells were observed.

To generate binary vector constructs for *Agrobacterium*-mediated transformation in Bienertia leaves, DNA fragments containing *BsCAβ1*, *BsCAβ2* or *BsCAβ2[C13,14S]*, together with both GFP and NOS terminator were digested with Xba1/EcoR1 restriction endonucleases and inserted into pBIB binary vector. Binary vector constructs shown in **Figure 1B** were constructed using Gateway cloning as described in Wimmer et al. (2019) with the primers listed in **Supplementary Table S1**. The PIP2A construct was ordered from the Nebenfuehr collection (Nelson et al., 2007).

### RNA quantitative real-time PCR analysis

Total RNA was prepared from leaf tissues which were harvested at three different developmental stages (early, intermediate and late stages) of 0.5−0.7 cm long Bienertia leaves (Koteyeva et al., 2016) using RNAiso Plus (Takara) with isopropanol. cDNA was synthesized using a high capacity cDNA reverse transcription kit (Applied Biosystems) with 2 µg of RNA. 200 ng of cDNA with Power SYBR Green PCR Master Mix (Applied Biosystems) was used for quantitative real-time PCR. All reactions were done in a triplicate. Elongation factor 1 was used as a reference gene (Lara et al., 2008). *P*-values were calculated by Student’s *t*-test at *P* < 0.05 and *P* < 0.01. Primer sequences were listed in **Supplementary Table S1**.

### *Agrobacterium*-mediated transformation by vacuum infiltration

For transient expression of constructs in leaf cells using *Agrobacterium*-mediated transformation, pBIB binary vectors containing *BsCAβ1:GFP*, *BsCAβ2:GFP* or *BsCAβ2[C13,14S]: GFP* introduced into *Agrobacterium tumefaciens* strain GV3101 or AGL1 by electroporation. Bacterial pellets were resuspended in 2× infiltration buffer (8.86 g/L Murashige and Skoog, 10% w/v sucrose, 20 mM MES (Duchefa Biochemie) and diluted to 0.4 of OD_600_. Finally, acetosyringone (Sigma-Aldrich) was added to the culture at 0.2 mM final concentration. The *Agrobacterium* solution was incubated for 1 h at room temperature. For *Agrobacterium*-mediated transformation by vacuum infiltration in leaf cells, 3−4 cm long branches of Bienertia plants were gathered. Leaves were pricked 8−13 times depending on the leaf size to increase the transformation efficiency. Prepared leaves were soaked in *Agrobacterium* solution. Vacuum infiltration was performed 3−4 times. Infiltrated leaves were incubated over 4 days under the low light (20−50 μmol*m^-2^*s^-1^) condition for the expression of the transgene.

### PEG transformation

Plasmids purified using Qiagen columns were introduced into Arabidopsis protoplasts using the polyethylene glycol (PEG)-mediated transformation (Kim et al., 2001; Jin et al., 2001) and into Bienertia protoplasts following the procedure described in Wimmer et al. (2017). Plasmids purified using Qiagen columns were introduced into Arabidopsis protoplasts using the polyethylene glycol (PEG)-mediated transformation (Kim et al., 2001; Jin et al., 2001) and Bienertia protoplasts following the procedure of Wimmer et al. (2017). Generally, 3- to 4-week-old Arabidopsis leaf tissues (5 g) were cut into small pieces (5 to 10 mm2) and incubated in 50 mL of enzyme solution [0.25% Macerozyme (Yakult Honsha Co., Ltd., Tokyo, Japan) R-10, 1.0% Cellulase (Yakult Honsha Co., Ltd.) R-10, 400 mM mannitol, 8 mM CaCl_2_ and 5 mM Mes-KOH, pH 5.6] at 22°C for 5 h with gentle agitation. Then, the suspension was filtered through 100-μm mesh and centrifuged at 46 x g for 5 min. The pelleted protoplasts were resuspended in 5 to 10 mL of W5 solution (154 mM NaCl, 125 mM CaCl_2_, 5 mM KCl, 5 mM glucose, and 1.5 mM Mes-KOH, pH 5.6), overlaid on top of 20 mL of 21% sucrose, and again centrifuged at 78 x g for 10 min. The intact protoplasts were collected at the interface and resuspended in W5 solution. To transform DNA into protoplasts, purified plasmid and PEG were added to protoplasts resuspended in MaMg (0.4 M Mannitol (w/v), 15mM MgCl_2_ (w/v), 0.1% (w/v) MES, adjust the pH to 5.6 with 1 M KOH), mixed gently and incubated at 22°C in the dark. Similarly, Bienertia mature leaves were sliced and incubated in the enzyme solution as in the case of Arabidopsis leaf tissue at 35 °C in a water bath for 1 h. The mixture was centrifuged at 51 x g for 1 min and the pellet was resuspended in 20% (w/v) sucrose + glycine-betaine. Intact protoplasts on top of the sucrose cushion were resuspended in glycine-betaine buffer (5 mM MES-NaOH, pH 5.7, 10 mM CaCl_2_, glycine-betaine) and centrifuged at 51 x g for 1 min. The pellet containing protoplasts were resuspend in glycine-betaine buffer for transformation with plasmid DNA. The concentration of glycine-betaine in the buffers for protoplast isolation was adjusted to match the internal osmolyte concentration of Bienertia chlorenchyma cells. Since the internal osmolarity varies significantly between experiments (ranging from approximately 600 to 1500 mOsmol), particularly following watering, the exact concentration of glycine-betaine was determined individually for each experiment. The internal osmolarity was measured immediately before protoplast isolation using a vapor pressure osmometer (Vapro 5520, Germany). The glycine-betaine concentration was then dynamically adjusted to ensure osmotic balance between the buffer and the protoplasts. Images of GFP fused proteins in transformed protoplasts were taken by a fluorescence microscope at 24 h after PEG-transformation (Zeiss Axio Plan 2) (Lee et al., 2008; Jin et al., 2001; Kwon et al., 2018). Total proteins were extracted from protoplasts after taking images.

### Tobacco infiltration

Agrobacteria transformed with different *BsCAβ1* and *BsCAβ2* constructs together with silencing suppressor p38 driven by the CsMV promoter were cultured in the LB medium at 28°C with two antibiotics, suspended cells were re-suspended in infiltration buffer (10 mM MES-KOH, pH 5.6, 10 mM MgSO_4_) at a concentration of 0.8 of OD at 600 nm. Subsequently, 400 μM acetosyringone was added into the re-suspended cells and incubated for 1 h at room temperature. Next, different combination of Agrobacteria harboring the binary constructs of *BsCAβ1* and *BsCAβ2* together with Agrobacteria harboring p38 were infiltrated into N. benthamiana leaves using a 1 mL syringe without a needle. After 3 days post-infiltration, leaves observed under a confocal microscopy to examine the GFP signal. In parallel, leaves samples were harvested for Western blot analysis to confirm the expression of proteins.

### Subcellular fractionation and western blot analysis

From Arabidopsis protoplasts, protein extracts were prepared as described (Lee et al., 2018; Ahn et al., 2017). Briefly, protoplasts were resuspended in cell lysis buffer followed by a brief sonication and centrifugation at 3,000 × g for 10 min to remove debris. Supernatants were treated with 1% Triton X-100 (v/v) or 0.1 M Na_2_CO_3_ (pH 11.5) and further incubated on ice for 30 min followed by ultracentrifugation at 150,000 × g for 1 h. Supernatant (soluble) and pellet (membrane) fractions were collected separately and analyzed by western blotting.

For preparation of protein extracts from leaf tissues of *Bienertia sinuspersici*, leaves 4 days after *Agrobacterium*-mediated infiltration were squeezed to release cellular contents into 300 µL buffer (50 mM Tris-HCl (pH 7.5), 150 mM NaCl, 3 mM MgCl_2_, 1 mM EDTA, 2 mM DTT and protease inhibitor cocktail) and the leaf exudates were centrifuged at 3,000 × g for 10 min to remove debris. Supernatant was collected and sonicated briefly. The protein extracts were centrifuged again at 18,300 × g for 10 min. Supernatant was treated with 1% Triton X-100 or 0.1 M Na_2_CO_3_ (pH 11.5) and further incubated on ice for 30 min followed by ultracentrifugation at 150,000 × g for 1 h. Soluble and pellet fractions were collected separately and analyzed by western blotting.

For western blot analysis, mouse anti-GFP (Clontech, 1:1,000 dilution), rabbit anti-VSR (1:1,000 dilution) (Kang et al., 2012), rabbit anti-Arabidopsis aleurain-like protein (AALP; 1:3,000 dilution) (Lee et al., 2006) antibodies were used. Images were captured by an LAS4000 image analyzer (Fujifilm).

### Blue native-polyacrylamide gel electrophoresis

*Agrobacterium*-mediated transformed Bienertia leaves were squeezed to release cellular contents into buffer (50 mM HEPES, pH 8.0, 15% w/v Glycerol, 0.1% Triton X-100 (v/v) and protease inhibitor cocktail). Leaf exudates were incubated on ice for 10 min followed by centrifugation at 3,000 × g for 10 min. Supernatant was sonicated briefly and centrifuged at 18,300 × g for 10 min, followed by ultracentrifugation at 100,000 × g for 10 min. The supernatant was collected and loaded onto precast 4 to 16% gradient gels (Invitrogen) (Yoo et al., 2016). A cathode (15 mM Bis-Tris, pH 7.2, 50 mM Tricine, 0.002% Coomassie brilliant blue G-250) and an anode buffer (50 mM Bis-Tris, pH 7.2) were used. BN-PAGE was performed at 4°C and the proteins were analyzed by western blotting using rabbit anti-GFP (1:1,000 dilution) (Bio-Application, Korea) antibody.

### *In vivo* imaging

All images were acquired by a LSM 900 confocal laser scanning microscope. The filter set had an excitation wavelength/spectral detection bandwidth of 488 to 530 nm for GFP and 560 to 615 nm for FM4-64. To measure relative signal intensity, the mean pixel values of cytosolic and adjacent PM were quantified separately b using ImageJ, and the ratio of their values was calculated.

## Results

### Transcript levels of *BsCAβ1* and *BsCAβ2* increase along with maturation of leaf in *Bienertia sinuspersici*

**Figure 2:**
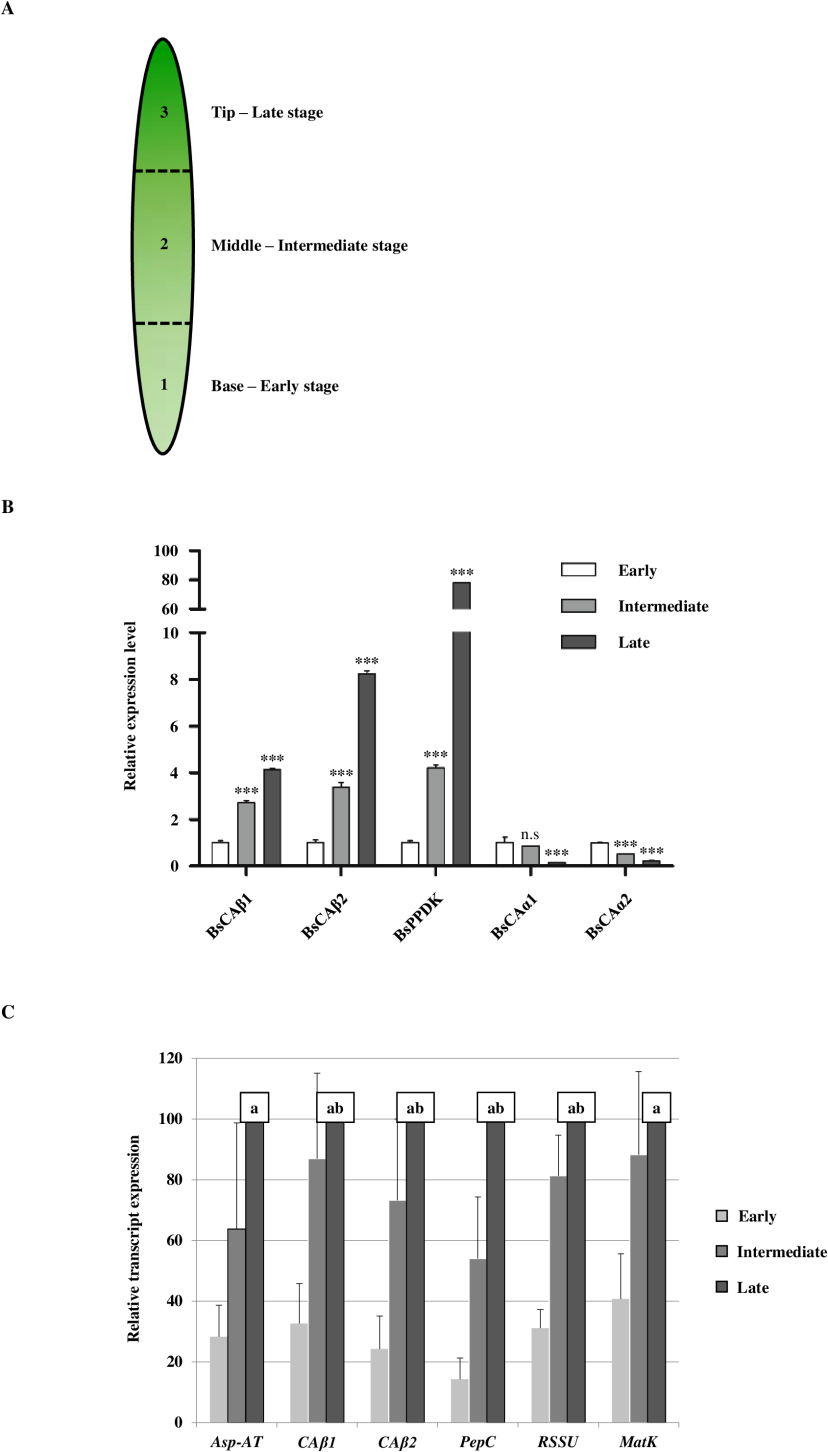
The expression of β-type carbonic anhydrase genes of *Bienertia sinuspersici* is gradually induced along with maturation of the leaf. **(A)** Schematic representation of the developmental stages of *Bienertia sinuspersici* leaf development. Bienertia has a unique biochemical and structural development along a longitudinal leaf gradient in 0.5−0.7 cm long young leaves. Over 1 cm, all cells in the leaf are at the mature (late) stage. The representative stages are marked along the leaf. **(B)** Transcript levels of α- and β-type *CAs* and *PPDK* of Bienertia depending on the leaf developmental stages. Total RNAs were extracted from leaf tissues at the indicated developmental stages and analyzed by qRT-PCR. *P*-values were calculated by Student’s *t*-test at *P* < 0.05 and *P* < 0.01. N = 3. **(C)** Transcript levels of β-type CAs in comparison to the C_4_ and C_3_ photosynthesis-related genes in Bienertia. Expression was normalized to the expression of elongation factor 1. Significant differences (2-sided ratio paired *t*-test, p < 0.05) are indicated with the letters a (significant difference between early and late stage) and b (significant difference between early and intermediate stage). N = 3. *** means the significance at P < 0.001 and n.s means not significant.

In an effort to get insight into the mechanism of the single cell C_4_ system in Bienertia, we identified genes related to the C_4_ photosynthesis in Bienertia. First we focused on carbonic anhydrases that catalyze the conversion of CO_2_ to HCO_3_- as part of the CO_2_-concentrating mechanism (CCM). These four CAs were grouped into two different subfamilies, α- and β-subfamilies. According to their classification, these CAs were named *BsCAα1*, *BsCAα2*, *BsCAβ1* and *BsCAβ2* (**Supplementary Figure S1**), and the mRNA sequences were submitted to GenBank under the accession numbers from MK674489 to MK674492. We first examined the expression of these *BsCAs* at three different developmental stages: early, intermediate and late stages (**Figure 2A**). A previous study provided a criterion on the developmental stage of leaves according to the maturation of dimorphic chloroplasts, which are the basis of the single cell C_4_ system in Bienertia (Koteyeva et al., 2016). The top quarter, middle half and bottom quarter of a leaf show the late, intermediate and early stages, respectively, in the leaf development (**Figure 2A**). First, we performed qRT-PCR to examine the expression pattern of these *BsCAs*. We also identified a gene named *BsPPDK* (GenBank accession number: MK674493) that encodes a polypeptide with a high degree of homology to pyruvate, phosphate dikinase (PPDK), a key enzyme in the C_4_ photosynthetic system and it was included in the qRT-PCR analysis as a control to compare the expression pattern of *BsCAs* with that of genes involved in the C_4_ photosynthesis in Bienertia. Both *BsCAβ1* and *BsCAβ2* showed strong induction in the expression along with the maturation of the leaf (**Figure 2B**). Moreover, this type of the expression pattern was similar to that of *BsPPDK* although *BsPPDK* showed much higher degree of induction at the late stage compared to the two *BsCAβs* (**Figure 2B**). By contrast, the two *BsCAαs* were highly expressed at the early stage and greatly reduced in the expression to much lower levels at the intermediate and late stages of the leaf (**Figure 2B**). The expression patterns of *BsCAB1* and *2* correlate well with the expression pattern of *BsPPDK*, a known key enzyme of the C_4_ carbon concentrating mechanism, raising the possibility that *BsCAβs* are involved in the C_4_ photosynthesis. In the C_4_ photosynthesis, β-type CAs are known to play a key role in CCM by converting CO_2_ to HCO_3_-. To further test this idea, we compared the expression pattern of *BsCAβ1* and *BsCAβ2* with other the C_4_ and C_3_ photosynthesis-related genes. The expression patterns of BsCAB1 and 2 correlate well with the expression pattern of BsPPDK, a known key enzyme of the C4 carbon concentrating mechanism, raising the possibility that BsCAβs are involved in the C4 photosynthesis. In the C4 photosynthesis, β-type CAs are known to play a key role in CCM by converting CO_2_ to HCO3-. The expression patterns of BsCAB1 and 2 correlate well with the expression pattern of BsPPDK, a known key enzyme of the C4 carbon concentrating mechanism, raising the possibility that BsCAβs are involved in the C4 photosynthesis. In the C4 photosynthesis, β-type CAs are known to play a key role in CCM by converting CO_2_ to HCO3-. To further test this idea, we compared the expression pattern of BsCAβ1 and BsCAβ2 with other the C4 and C3 photosynthesis-related genes. Aspartate aminotransferase (Asp-AT), a key enzyme for carbon metabolism of the C4 photosynthesis, converts oxaloacetate to aspartate. This organic acid further takes part in several processes including carbon fixation. Together with Asp-AT, phosphoenolpyruvate carboxylase (PEPC), also an essential component of the C4 photosynthesis, catalyzes the β-carboxylation of PEP to produce oxaloacetate (OAA), a four-carbon organic acid. Rubisco which is the most abundant protein in plants catalyzes the initial CO_2_ fixation of photosynthesis as one of the major factor of plant productivity (Parry et al., 2013). Plastid-encoded maturase K (MatK) involved in intron-splicing in chloroplasts. This RNA processing factor plays a vital role in photosynthetic competency of the chloroplast and also the survival of plant cells (Hirao et al., 2009). In this study, we checked the transcript level of four genes including: BsAsp-AT, BsPEPC, BsRSSU, and BsMatK. The expression pattern of the two *BsCAβs* closely resembled the two C_4_-related genes as well as *BsRSSU* and *BsMatK* (**Figure 2C**).

### BsCAβ1:GFP and BsCAβ2:GFP mainly localize to the cytosol and plasma membrane, respectively, in Bienertia leaf cells and two heterologous plants

**Figure 3:**
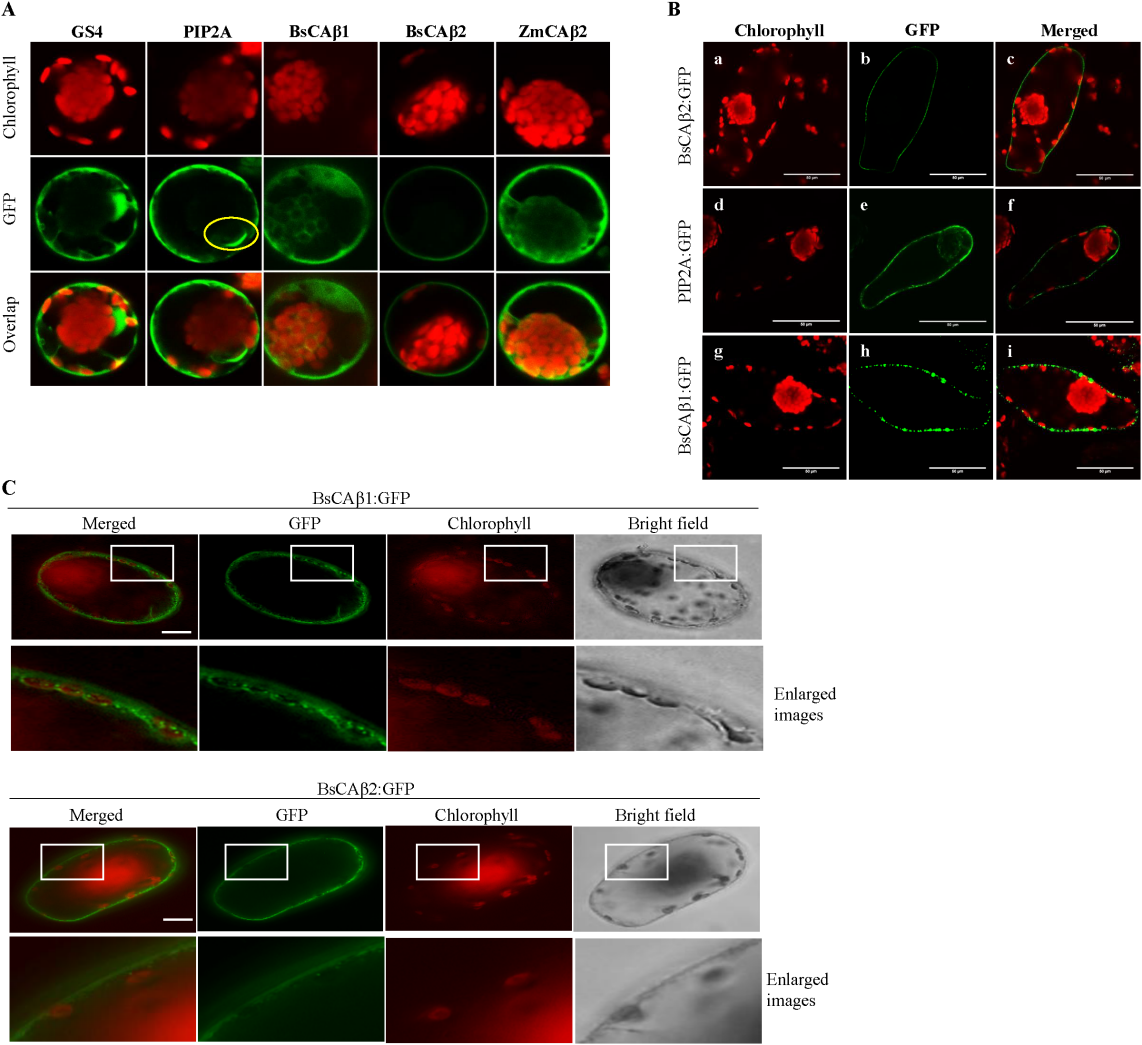
Subcellular localization of BsCAβs:GFP in Bienertia cells. **(A)** *In vivo* localization of BsCAβs:GFP in Bienertia protoplasts. Protoplasts were transformed with BsCAβs:GFP and their localization was determined 24 h after PEG transformation. Confocal images showing chlorophyll autofluorescence (upper row), GFP (middle row) and merged images (lower row). GS4, GFP alone; PIP2A, plasma membrane aquaporin 2A; ZmCAβ2, β-carbonic anhydrase 2 from Zea maize. Yellow circle indicates location of the nucleus. Magnification in all images = 600×. **(B)** *In vivo* localization of BsCAβs:GFP in Bienertia leaf chlorenchyma cells using *Agrobacterium*-mediated transformation. Confocal images showing the red chlorophyll autofluorescence (first column), the GFP channel (second column) and an overlay of both channels (third column). a−c, BsCAβ2:GFP; d−f, PIP2A:GFP (plasma membrane intrinsic aquaporin 2A); g−I, BsCAβ1:GFP. All scale bars = 50 µm. **(C)** Localization of BsCAβ1:GFP and BsCAb2:GFP in Bienertia leaf cells. Intact Bienertia leaves were transformed by *Agrobacterium*-mediated transformation using vacuum infiltration. GFP patterns were observed 4 days after infiltration. The enlarged images of the boxed areas in the first and third lows are shown in the second and fourth lows, respectively. Green and red signals represented GFP and autofluorecence of chlorophyll, respectively. Scale bar = 50 μm.

To gain insights into the physiological roles of BsCAβs, we examined their subcellular localization. CAs are found in various subcellular locations and their physiological roles are closely related to their subcellular localization (Moroney et al., 2001; Fabre et al., 2007; Razzak et al., 2019). We analyzed the amino acid sequence of BsCAβ1 and BsCAβ2 using the prediction tool TMHMM (http://www.cbs.dtu.dk/services/TMHMM/) for any possible targeting signals and found that both BsCAβ1 and BsCAβ2 did not contain any specific localization signals, indicating cytosolic localization. Thus, we examined the localization experimentally. The C-terminal GFP-fused forms of *BsCAs* (*BsCAβ1:GFP* and *BsCAβ2:GFP*) were firstly transiently overexpressed in protoplasts prepared from mature Bienertia leaves following a protocol that was established to analyze protein import into Bienertia chloroplasts (Wimmer et al., 2017, 2019). BsCAβ2:GFP showed a clear ring pattern which was mostly similar to the pattern observed for PIP2A, a plasma membrane (PM) aquaporin (Lee et al., 2009). However, PIP2A also showed some GFP-label around the nuclear envelope (yellow circle) which was not observed for BsCAβ2:GFP (**Figure 3A**). In contrast, BsCAβ1:GFP showed a diffuse pattern which resembled most closely the pattern observed for the GFP alone (GS4); GFP signal appeared to originate from the peripheral cytoplasm, the cytoplasm surrounding the central chloroplasts as well as from the interconnecting cytoplasmic channels. For comparison, we also analyzed maize βCA2 (ZmCAβ2), which showed a similar cytoplasmic distribution as BsCAβ1:GFP and the GS4 cytosolic control. Localization was further analyzed in intact leaf cells using an *Agrobacterium*-mediated transient transformation protocol (Wimmer et al., 2019). Again, BsCAβ2:GFP produced a very clear ring-type pattern (**Figures 3B, C**) and the GFP distribution was very similar to the distribution of PIP2A:GFP (**Figures 3A**), confirming the PM localization. In contrast to BsCAβ2:GFP, BsCAβ1:GFP produced a punctate-staining pattern instead of a diffuse cytosolic pattern. The puncta of BsCAβ1:GFP appeared to localize closely to the PM (**Figures 3A, B**). We got a close look of the localization by enlarging the image from protoplasts (**Figure 3C**). BsCAβ1:GFP was observed around the peripheral chloroplasts as well as in the cytosol nearby the chloroplasts. In contrast, BsCAβ2:GFP was not detected around chloroplasts and instead gave a pattern of a linear line, an indication of the PM localization.

To further confirm the localization of these two CAs, we utilized heterologous systems, *Arabidopsis thaliana* and *Nicotiana benthamiana*. First, BsCAβ1:GFP or BsCAβ2:GFP was introduced into Arabidopsis protoplasts by PEG-mediated transformation (Kim et al., 2001; Jin et al., 2001). BsCAβ1:GFP produced a diffuse pattern in the cytosol in Arabidopsis protoplasts as observed in Bienertia (**Figure 1A**). In contrast, BsCAβ2 showed a clear PM localization as observed in Bienertia. Next, we expressed these two constructs transiently in *N. benthamiana* leaves. BsCAβ1:GFP displayed the mainly punctuated pattern together with some large clump in the cytosol (**Figure 1B**). By contrast, BsCAβ2:GFP produced a ring pattern, an indication of the PM localization, together with a few speckles. In addition, the GFP signal of BsCAβ2 overlapped to FM4-64, a PM marker that again confirmed the localization of BsCAβ2:GFP (**Figure 1B**). Thus, the *in vivo* localization studying in three different plant species indicate that BsCAβ1 and BsCAβ2 differentially localize to the cytosol and plasma membrane, respectively.

**Figure 4:**
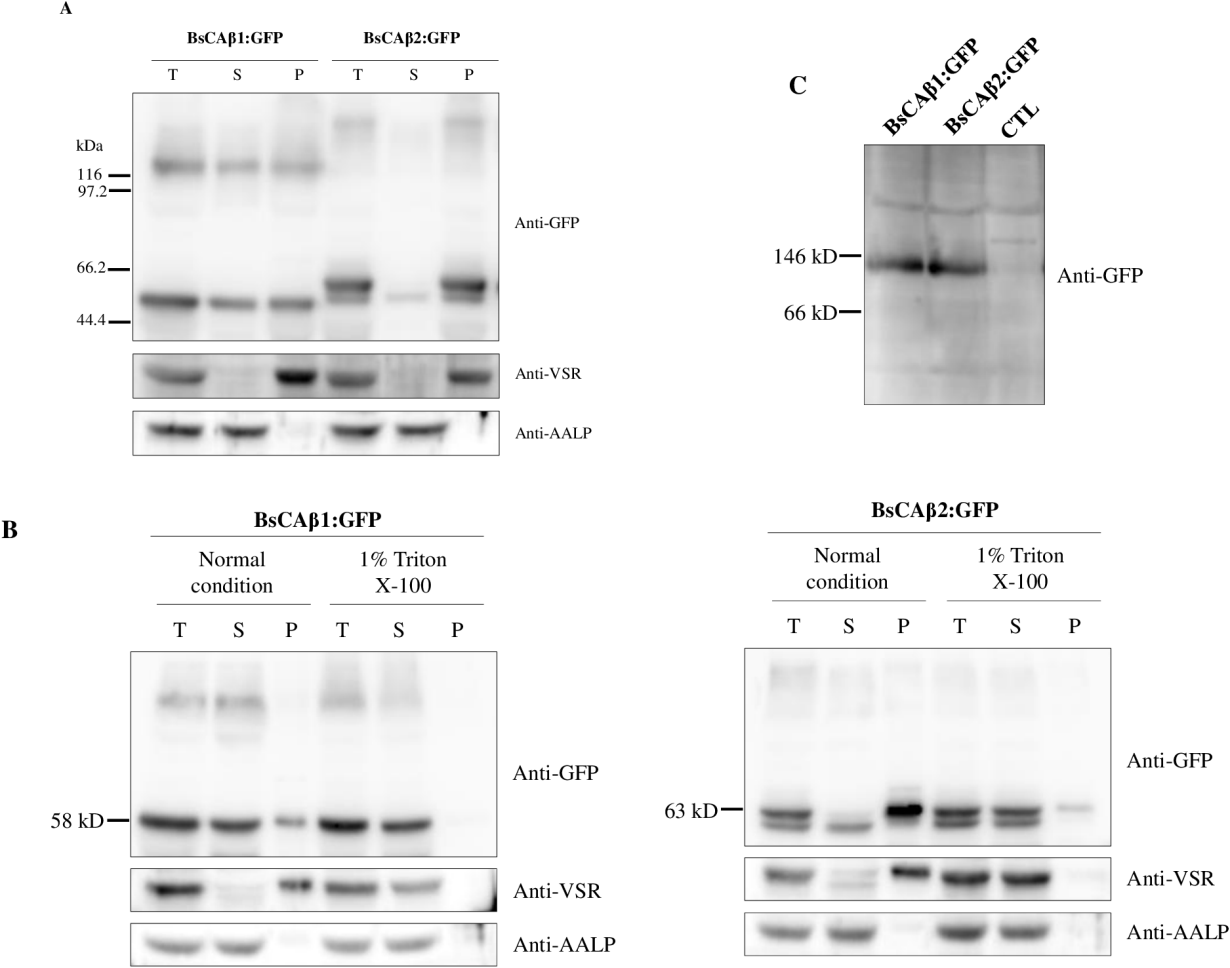
Biochemical analysis for the localization and dimer formation of BsCAβ1:GFP and BsCAβ2:GFP in Bienertia. **(A)** Subcellular fractionation of BsCAβs:GFP in Bienertia leaf cell. Total protein extracts from Bienertia leaves transformed with GFP-tagged BsCAβs via *Agrobacterium*-mediated infiltration were separated into soluble and pellet fractions by ultracentrifugation and these fractions were separated by 10% SDS-PAGE and analyzed by western blotting using anti-GFP antibody. Anti-VSR and anti-AALP antibodies were used as controls for endogenous membrane and soluble proteins, respectively. T, total fraction; S, soluble fraction; P, pellet fraction. **(B)** Solubilization of BsCAβ2:GFP using Triton X-100. Total protein extracts were treated with 1% Triton X-100 and separated into soluble and pellet fractions by ultracentrifugation. These fractions were separated by 10% SDS-PAGE and analyzed by western blotting using anti-GFP, anti-VSR and anti-AALP antibodies. **(C)** Dimer formation of BsCAβ1:GFP and BsCAβ2:GFP. Total protein extracts from Bienertia leaf tissues transformed with *BsCAβs:GFP* were separated by BN-PAGE followed by western blot analysis using anti-GFP antibody. CTL; non-transformed leaves.

To corroborate this finding at the biochemical level, total protein extracts from Bienertia leaves transformed with *BsCAβ1:GFP* or *BsCAβ2:GFP* by *Agrobacterium*-mediated infiltration were separated into soluble and membrane fractions by ultracentrifugation and these fractions were analyzed by western blotting using an anti-GFP antibody. BsCAβ1:GFP was detected equally at both soluble and membrane fractions at the 58 kD position, indicating that BsCAβ1:GFP exists as both soluble and membrane-associated forms (**Figure 4A**). BsCAβ1:GFP also produced another band at about 116 kD position that was also detected at equal level in both fractions (**Figure 4A**), indicating that BsCAβ1:GFP exists as multimers such as dimers. β-CAs largely are known to exist and function as dimers or higher-order oligomeric forms (Aggarwal et al., 2015). Often multimers are not fully disassociated even after boiling of proteins in the SDS sample buffer containing 10% SDS and 2 mM DTT (Yoo et al., 2016). In the case of BsCAβ2:GFP, it was detected as two bands; a major band at the position of 63 kD and a minor band just below it, which was in contrast to the single band of BsCAβ1:GFP. Upon separation by ultracentrifugation, the lower minor band was detected in the soluble fraction whereas the upper major band was detected in the pellet fraction, indicating that the majority of BsCAβ2 localizes to the membrane in accordance with the *in vivo* localization observed in both Bienertia protoplasts, and isolated Bienertia cells. As in the case of BsCAβ1:GFP, BsCAβ2:GFP also produced a higher molecular weight form at around 130 kD position in the pellet fraction (**Figure 4A**). These results suggest that BsCAβ2:GFP largely localizes to the PM and exists as dimer or multimers. The fractionation of protein extracts were confirmed using anti-VSR (vacuolar sorting receptor) and anti-AALP(aleurain-like protease) antibodies (**Figure 4A**). VSR and AALP that localize to the prevacuolar compartment and vacuole, respectively, can be used to represent membrane and soluble proteins, respectively.

To further confirm that BsCAβ2:GFP localizes to the membranes, total protein extracts from Bienertia was treated with Triton X-100 and separated into soluble and pellet fractions by ultracentrifugation. As a control, protein extracts of BsCAβ1:GFP was also included in the fractionation experiments. These fractions were again analyzed by western blotting using anti-GFP antibody. Upon treatment with Triton X-100, the minor portion detected in the pellet fraction was detected in the soluble fraction (**Figure 4B**), indicating that a minor portion of BsCAβ1:GFP was associated with membranes. In the case of BsCAβ2:GFP, most of the proteins were detected in the soluble fractions with still a minor portion in the membrane fraction, confirming that the large portion of BsCAβ2:GFP localizes to membranes. Similar results were obtained with both BsCAβ1:GFP and BsCAβ2:GFP when expressed in *N. benthamiana* (**Supplementary Figure S2**). Here one noticeable difference was that the higher molecular weight form at 130 kD was more prominent in *N. benthamiana* than in Bienertia. This difference is not fully understood at this moment.

Western blot analysis in **Figure 4A** revealed that both BsCAβ1:GFP BsCAβ2:GFP were detected as multimers. Thus, we examined the complex formation of BsCAβ1 and BsCAβ2. Previous studies showed that the dimer is the basic structural unit of β-type CAs (Rowlett, 2010; Kimber and Pai, 2000). We performed Blue Native-PAGE (BN-PAGE) (Eubel et al., 2005; Reisinger and Eichacker, 2008; Yoo et al., 2016). Total protein extracts were prepared from leaf tissues of Bienertia expressing *BsCAβ1:GFP* or *BsCAβ2:GFP* after *Agrobacterium*-mediated infiltration. After BN-PAGE, proteins were analyzed by western blotting using anti-GFP antibody. Both BsCAβ1:GFP and BsCAβ2:GFP were detected at the position of approximately 120 kD (**Figure 4C**), indicating that both of them exist as a dimeric form *in vivo*, similar to β-type CAs in other organisms (Atkins, 1974; Atkins et al., 1972).

### BsCAβ2 potentially localizes to the PM via posttranslational modification

**Figure 5:**
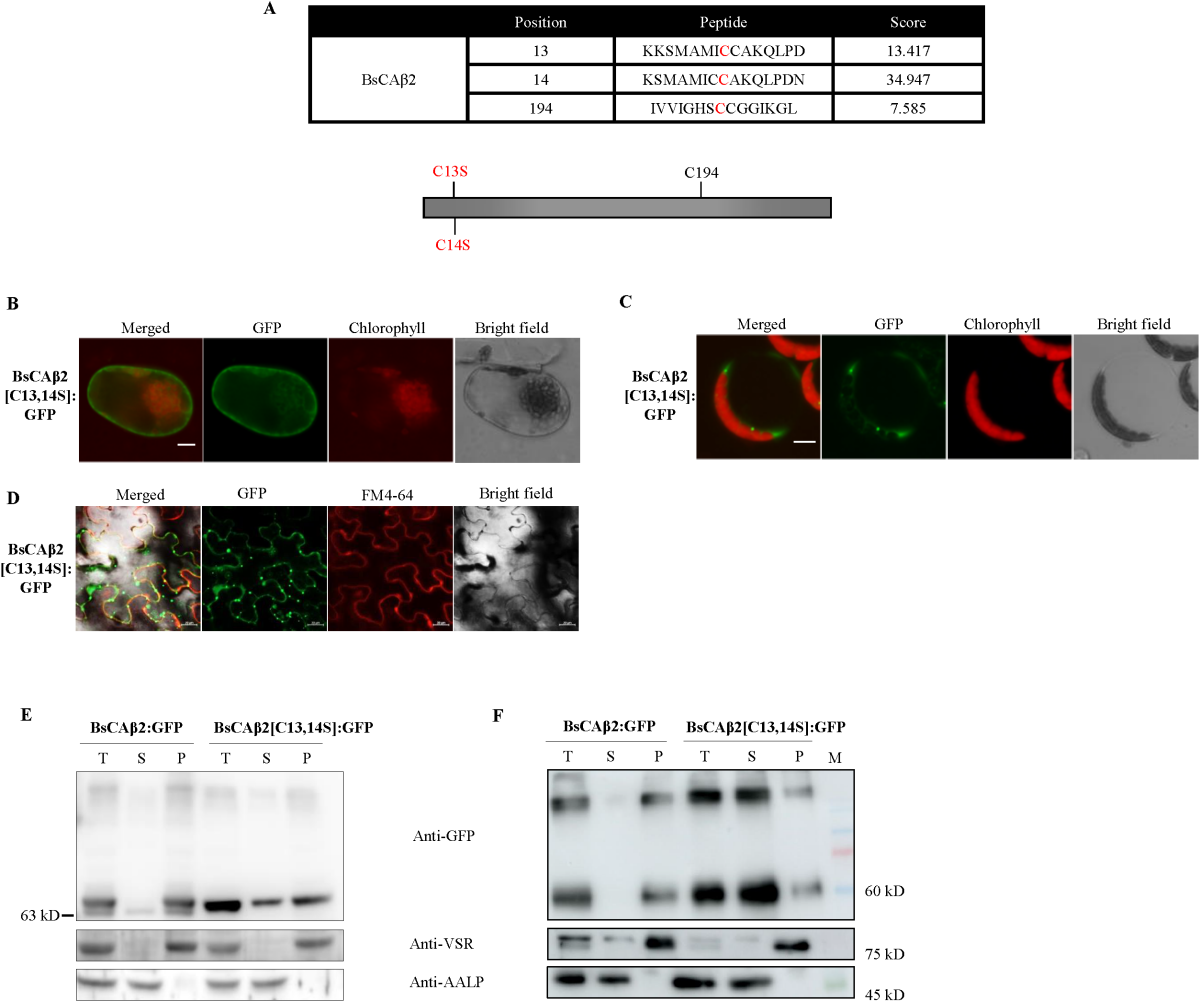
BsCAβ2 is subject to posttranslational modification inBienertia as well as two heterologous systems. **(A)** Sequence analysis by *CSS-PALM*. Putative palmitoylation sites were predicted by CSS-PALM 4.0 set with high threshold. 3 cysteine residues (C13, C14 and C194) were predicted as palmitoylation sites. High score residues (C13 and C14) at the N-terminal region were substituted with serines and indicated in red. **(B-D)** Localization of BsCAβ2[C13,14S]: GFP. *BsCAβ2[C13,14S]: GFP* was transformed into Bienertia leaf cells by *Agrobacterium*-mediated infiltration **(B)** Arabidopsis protoplast by PEG-mediated transformation **(C)** or leaf cells of *N. benthamiana* transformed by Agrobacterium-mediated infiltration **(D)**. GFP signals in Bienertia, Arabidopsis protoplasts and N. benthamiana were observed 4 days, 24 h and 3 after transformation, respectively. *N. benthamiana* leaf cells were stained with FM4-64 at 5 min before observation. GFP and chlorophyll autofluorescence are shown in green and red signals, respectively. Also, in N. benthamiana leaf cells, FM4-64 was shown in red channel. Scale bar = 20 μm. **(E, F)** Subcellular fractionation of BsCAβ2[C13,14S]: GFP. Total protein extracts from transformed Bienertia **(E)** and leaf tissues of N. benthamiana **(F)** were separated into soluble and membrane fractions by ultracentrifugation and these fractions were analyzed by western blot analysis using anti-GFP antibody. Endogenous VSR and AALP were used as representative of membrane and soluble proteins, respectively. T, total fraction; S, soluble fraction; P, pellet fraction.

The plasma membrane localization of BsCAβ2 prompted us to examine the primary sequence of BsCAβ2. When analyzed using the prediction tool TMHMM, it did not contain any transmembrane domain. This finding together with the fact that BsCAβ2:GFP yielded a doublet band by western blot analysis (**Figure 4**) raised the possibility of posttranslational modification (PTM) as the underlying mechanism for the PM localization of BsCAβ2. Often, proteins can be anchored to membranes by a lipid moiety added after translation (Nadolski and Linder, 2007). Therefore, BsCAβ2 was analyzed for the possibility of posttranslational lipid-based modification using the *CSS-PALM* prediction tool (Zhou et al., 2006). BsCAβ2 was predicted to have potential palmitoylation sites (C13 and C14) at the N-terminal region (**Figure 5A**). Palmitoylation is a reversible posttranslational modification (PTM) that attaches a 16-carbon palmitate to cysteine residues of proteins (Hemsley and Grierson, 2008; Hurst and Hemsley, 2015; Rocks et al., 2010). The palmitoylation promotes soluble proteins to be associated with membranes (Blaskovic et al., 2013). In addition, the reversible nature of lipidation allows dynamic control of protein association to membranes in a signal-dependent manner (Hurst and Hemsley, 2015).

To test whether BsCAβ2 is subject to PTM, we constructed a mutant construct *BsCAβ2[C13,14S]* in which C13 and C14 residues with high scores of palmitoylation were substituted with serine residues (**Figure 5A**). *BsCAβ2[C13,14S]* was then fused to GFP, and the resulting construct was expressed in both protoplast of Bienertia, Arabidopsis, and also in tobacco leaves. BsCAβ2[C13,14S]: GFP showed a mainly cytosolic pattern in both Bienertia and Arabidopsis protoplasts when examined by a fluorescence microscope (**Figures 5B, C**). Similarly, majority of BsCAβ2[C13,14S]: GFP in tobacco leaves exhibited the punctuate-staining pattern with a minor portion of GFP signal overlapping with FM4-64 dye (**Figure 5D**). Together these results indicate that the cysteine residues are critical for localization of BsCAβ2 to the PM. Moreover, these results strongly suggest that palmitoylation at C13 and/or C14 is the PTM responsible for anchoring BsCAβ2 to the PM.

To obtain supporting evidence for this finding, the localization of BsCAβ2[C13,14S]: GFP was examined at the biochemical level. Protein extracts from Bienertia protoplasts were separated into soluble and membrane fractions by ultracentrifugation and these fractions were subjected to Western blot analysis. In Bienertia, BsCAβ2[C13,14S]: GFP showed a different pattern from wild-type BsCAβ2:GFP (**Figure 5E**); unlike BsCAβ2:GFP, BsCAβ2[C13,14S]: GFP yielded a single band and a significant portion of it was detected in the soluble fraction (**Figure 5E**). However, still a major portion was detected in the membrane fraction, indicating that BsCAβ2[C13,14S] uses another mechanism for membrane association in Bienertia. To confirm this finding, total protein extracts from leaf tissues of *N. benthamiana* expressing BsCAβ2[C13,14S]: GFP were also separated into soluble and pellet fractions and these fractions were analyzed by western blotting using anti-GFP antibody. The major portion of BsCAβ2[C13,14S] was mainly detected in the soluble fraction with a very minor portion in the pellet fraction (**Figure 5F**), further supporting the idea that palmitoylation plays a critical role in the PM anchoring. One noticeable feature was that BsCAβ2:GFP in *N. benthamiana* was detected as a single band in contrast to Bienertia, indicating that the modification occurs more efficiently in leaf cells of *N. benthamiana* than in protoplasts of Bienertia.

### The posttranslational modification of BsCAβ2 may occur at the plasma membrane

**Figure 6:**
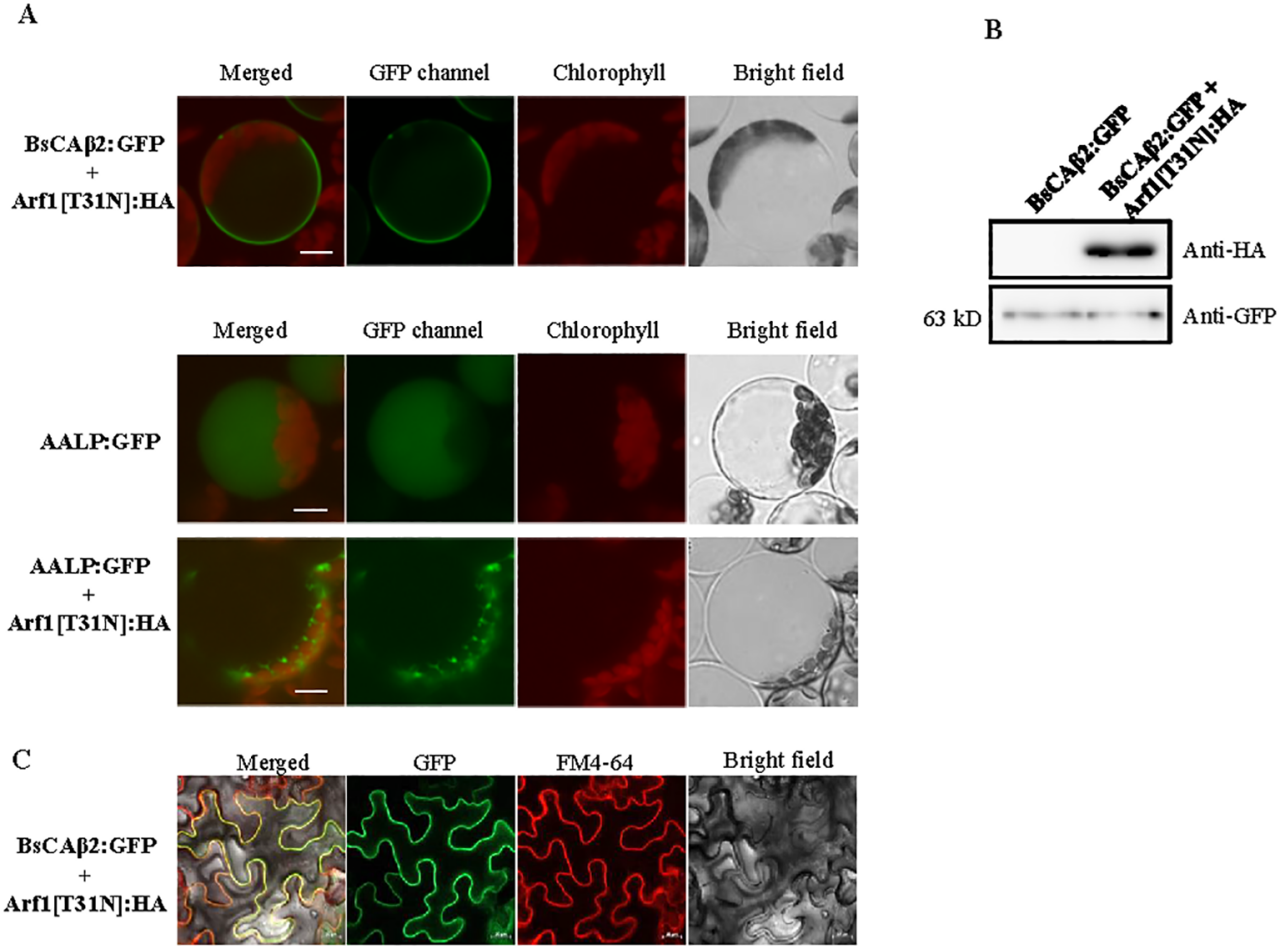
Posttranslation modification of BsCAβ2 occurs at the plasma membrane. **(A)** Localization of BsCAβ2:GFP in the presence of Arf1[T31N] in Arabidopsis protoplasts. BsCAβ2:GFP or AALP: GFP were introduced into protoplasts together with or without Arf1[T31N]: HA and the localization was examined 24/72 h after PEG-mediated transformation. GFP and autofluorescence of chlorophyll are shown in green and red signals, respectively. Scale bars = 20 μm. **(B)** Expression of BsCAβ2:GFP and Arf1[T31N]: HA. Total protein extracts from protoplasts were analyzed by western blotting using anti-GFP and anti-HA antibodies. **(C)** *In vivo* localization of BsCAβ2:GFP in leaf cells of N. benthamiana. BsCAβ2:GFP together with Arf1[T31N] was transformed into leaf cells of N. benthamiana via Agrobacterium-mediated infiltration. The localization was examined 3 days post of infiltration. Leaf tissues were stained with FM4-64 5 min before observation. GFP and FM4-64 dye are shown in the green and red channels, respectively. Scale bars = 20 µm. A total of 30 cells were observed and a representative image is shown. Subcellular fractionation of BsCAβ2:GFP. Total protein extracts from infiltrated tobacco leaves were separated into soluble and membrane fractions by ultracentrifugation and these fractions were analyzed by western blot analysis using anti-GFP and anti-HA antibodies for BsCAb2:GFP and Arf1[T31N]: HA, respectively.

The palmitoylation, also known as S-acylation, of proteins requires S-acyl transferases (PATs) (Hemsley, 2017). Previous studies showed that multiple PATs exist in plants (Li et al., 2016). The majority of plant PATs localizes to the plasma membrane in contrast to the PATs of animals and yeast that are primarily found at the ER or Golgi apparatus (Batistic, 2012; Ohno et al., 2006). As an approach to elucidate where the PTM of BsCAβ2 occurs in plants, we examined the possibility of whether it as a lipid-modified form traffics from the ER to the PM through the Golgi apparatus. We reasoned here that if palmitoylation of BsCAβ2 occurs at the ER the membrane-localized BsCAβ2 would be transported to the PM via protein trafficking. To test this, we used a dominant negative mutant of Arf1 (Arf1[T31N]). Arf1[T31N] inhibits trafficking of protein from the ER to the Golgi (Dascher and Balch, 1994; Lee et al., 2002; Park et al., 2016). We constructed *Arf1[T31N]: HA* and co-expressed with *BsCAβ2:GFP* in Arabidopsis protoplasts. The localization of BsCAβ2:GFP was examined under the fluorescence microscope. In the presence of Arf1[T31N]: HA, BsCAβ2:GFP was still localized to the PM, indicating that the localization of BsCAβ2:GFP is not affected by Arf1[T31N]: HA (**Figure 6A**). As control for the effect of Arf1[T31N]: HA on trafficking from the ER, we examined the trafficking of AALP: GFP to the vacuole. AALP is the luminal protein of the vacuole and known to traffic from the ER to the vacuole through the Golgi apparatus (Sohn et al., 2003; Kang et al., 2012; Lee et al., 2017). AALP: GFP alone was transported to the vacuole (**Figure 6A**). However, in the presence of Arf1[T31N]: HA, AALP: GFP was not properly trafficked to the vacuole (**Figure 6A**), confirming the effect of Arf1[T31N]: HA on the trafficking from the ER. These results imply that localization of BsCAβ2:GFP to the PM does not require Arf1-dependent trafficking from the ER. To confirm the expression of these genes in protoplasts, we prepared total protein extracts from protoplasts and analyzed by western blotting using anti-GFP and anti-HA (**Figure 6B**). Both Arf1[T31N]: HA and BsCAβ2:GFP were detected at the expected position by anti-HA and anti-GFP antibodies, respectively, confirming that these genes were properly expressed in protoplasts. These results suggest that PTM of BsCAβ2 may occur at the PM. To further confirm this, we also tested targeting of BsCAβ2[C1,14S]: GFP in leaf cells of N*. benthamiana* after *Agrobacterium*-mediated infiltration. Again GFP image was mainly detected at the PM that overlapped to the signal of FM4-64 dye (**Figure 6C**), confirming that BsCAβ2:GFP is properly targeted to the PM in the presence of Arf1[T31N]: HA.

## Discussion

In this study, we investigated CAs of Bienertia at the molecular, biochemical and cellular levels. We identified two β-type CAs, *BsCAβ1* and *BsCAβ2*, and two α-type CAs, *BsCAα1* and *BsCAα2*, from RNA seq data of Bienertia. We focused on β-type CAs for their physiological roles. Previous studies showed that β-type CAs play a crucial role in CCM in plants with the C_4_ photosynthesis system (Badger et al., 1980; Giordano et al., 2005; Moroney et al., 1989). Expression studies revealed that transcript levels of *BsCAβ1* and *BsCAβ2* greatly increased along with maturation of Bienertia leaf whereas transcript levels of the two α-type CAs, *BsCAα1* and *BsCAα2* decreased (**Figure 2B**), showing that the α- and β-type CAs in Bienertia exhibit quite opposing expression patterns along with the development of leaf cells. Similarly, certain βCAs in other organisms such as Arabidopsis, and *Zea mays*, *Panicum virgatum*, *Gynandropsis gynandra*, *Setaria viridis*, and *Sorghum bicolor* are highly expressed in leaf tissues (DiMario et al., 2017). In Bienertia, the leaf cells are known to have different developmental stages depending on the position of the cells in the leaf; cells undergo maturation from cells performing the C_3_-type photosynthesis at the base to the cells performing the C_4_-type photosynthesis at the tip of leaf (Lara et al., 2008). Thus, the fact that *BsCAβs* are expressed at high levels at a later stage of leaf development raises the possibility that they are involved in the CCM of the C_4_ photosynthesis. Supporting this notion is that the C_4_ photosynthesis-related genes such as *BsPPDK* and *BsPEPC* showed similar expression patterns as *BsCAβs*.

CAs have been shown to localize to various subcellular locations such as chloroplasts, mitochondria, the cytosol and the plasma membrane (DiMario et al., 2017; Razzak et al., 2019). The differential localization is controlled by specific targeting signals such as transit peptide. The localization of CAs is critically related to their physiological roles. Since the initial CO_2_ fixation mechanism occurs in the cytosol of mesophyll cells in Kranz-type C_4_ photosynthetic organisms such as maize (Fouracre et al., 2014), CAs involved in a CCM should localize to the cytosol. We elucidated the localization of BsCAβs. Our localization study revealed that BsCAβ1 localizes to the cytosol and PM, whereas BsCAβ2 localizes exclusively to the PM although it does not contain any TMDs (**Figures 1A, B**, **3A**). In Arabidopsis, AtβCA4.1 localizes to the PM whereas the shorter form AtβCA4.2 generated by alternative splicing localizes to the cytosol (DiMario et al., 2016). The cytosol-localized BsCAβ1 might contribute to conversion of CO_2_ to HCO_3_- in the cytosol. However, it is not clear how BsCAβ2 localized to the plasma membrane plays a role in CCM. In Arabidopsis, PM-localized AtβCA4 is thought to play a role in CO_2_ sensing together with AtβCA1 (Hu et al., 2010). However, the expression level of *AtβCA4* is low in leaf cells, amounting to only 7% of that of AtβCA1 localized to chloroplasts (DiMario et al., 2016), indicating that the function of PM-localized BsCAβ2 may not be directly deduced from that of AtβCA4. One possibility is that in the cell with a big volume such as the cells in Bienertia leaf, it is critical to capture CO_2_ as soon as it enters into the cell to be used for photosynthesis. Thus, PM-localized BsCAβ2 is ideally position to convert CO_2_ to HCO_3_- as soon as it enters the cell. Consistent with this hypothesis, it has been proposed that in Bienertia pyruvate, phosphate dikinase (PPDK) localizes to peripheral chloroplasts near the PM and produces PEP from pyruvate (Offermann et al., 2011, 2015). Thus, HCO_3_- generated by plasma membrane-localized BsCAβ2 can be readily combined with PEP at the peripheral chloroplasts to make the C_4_ acid, oxaloacetate, which then diffuses to the central chloroplasts after conversion to aspartate. Alternatively, PM localization could also help to confine CA activity generally to the peripheral compartment and away from the central compartment. This could be important since CO_2_-release from C_4_ acids occurs within the mitochondria of the central compartment. The CO_2_ released then diffuses into the surrounding chloroplasts of the central compartment for final CO_2_ fixation by Rubisco. Therefore, significant CA activity in the central compartment could interfere with the efficiency of the CCM through re-hydration of CO_2_ to HCO_3_- which would effectively lower the CO_2_ availability for Rubisco in the central chloroplasts.

Membrane-localized proteins need to have a domain for membrane anchoring. The most common anchor is the transmembrane domain. BsCAβ2 localized to the PM in both Bienertia and Arabidopsis. However, BsCAβ2 does not have a TMD. In Arabidopsis, one form of β-type CA, AtβCA4, also localizes to the plasma membrane (Fabre et al., 2007). The nature of its PM localization is not known. In the case of BsCAβ2, we found that it has the potential palmitoylation sites at the N-terminal region. Furthermore, we provide convincing evidence that BsCAβ2 is subject to PTM, which is critical for membrane anchoring of BsCAβ2 to the plasma membrane. This conclusion is based on the fact that BsCAβ2[C13,14S] mutant cannot localize to the plasma membrane. Moreover, wild-type BsCAβ2:GFP has a slightly higher molecular mass than BsCAβ2[C13,14S]: GFP. We favor that the PTM is palmitoylation based on the prediction as well as the mutant analysis. Similarly, the long form of Arabidopsis AtβCA4 that localizes to the PM also predicted to contain palmitoylation sites such as C8, C12 and C13 when analyze by the *CSS-PALM* prediction tool (Zhou et al., 2006). By contrast, AtβCA4.2, the shorter form localized to the cytosol, does not contain the N-terminal region containing these cysteine residues (DiMario et al., 2017). Thus, N-terminal palmitoylation of beta type CAs is a mechanism for PM anchorage. However, we did not address the nature of the lipid moiety in this study. Palmitoylation is a reversible modification, which allows for dynamic regulation of protein association to membranes in a signal-dependent manner (Conibear and Davis, 2010). However, it is not clear whether the PTM of BsCAβ2 is dynamically regulated depending on certain cellular and/or environmental conditions. In addition, BsCAβ1 which was not subject to PTM also existed partially as a membrane-associated form. The nature of its membrane association is not clearly understood. When heterologously expressed in Arabidopsis, BsCAβ1 was multimedetected in the soluble fraction, indicating that in Bienertia BsCAβ1 may employ a unique mechanism for the membrane association.

The most important question for BsCAβ1 and BsCAβ2 is their physiological role. Based on various characteristic features of the two BsCAβs, we propose that they are involved in the C_4_ photosynthesis by playing a crucial role in the CCM. However, additional lines of evidence may be necessary to support this idea. Currently, with Bienertia, it is still not possible to generate transgenic plants or to obtain mutants with loss-of-function at these loci. A previous study with Arabidopsis showed that double knock-out mutants of βCA1 and βCA4 have higher density of stomata and display a defect in CO_2_-mediated regulation of stomatal movement under various CO_2_ concentrations (Hu et al., 2010). Overexpression of βCA1 and βCA4 enhanced water use efficiency (Hu et al., 2010). Also, the leaf size of *ca1ca4* double mutant plants was smaller than that of wild-type plants. At the moment, it is not clearly understood the mechanism underlying the increase in the biomass. A recent study revealed that overexpression of *FbβCA3* leads to an increase in amino acid production. The authors proposed that the higher levels of amino acids lead to the increase in biomass (Kando et al., 2022). Thus, one future direction of the research would be stably overexpressing these genes in Arabidopsis to examine the developmental morphology, the photosynthesis efficiency between the wild type, double mutant and overexpressed plants.

## Data Availability

Sequence data in this work can be found in GenBank under the following accession numbers: *BsCAα1*, MK674489; *BsCAα2*, MK674490; *BsCAβ1*, MK674491; *BsCAβ2*, MK674492; and *BsPPDK*, MK674493.

## Supplementary material

Supplementary Figure 1 Phylogenetic trees of carbonic anhydrases of Bienertia. Neighbor-Joining trees of α-type carbonic anhydrases **(A)** and β-type carbonic anhydrases **(B)** for *Bienertia sinuspersici* (red), compared to paralogous proteins of representative monocots and eudicots. All monocots and eudicots are marked as yellow circles and green triangles, respectively. These trees were computed using the Poisson correction method with pairwise deletion, and only > 50% bootstrap support are shown within 1000 replicates. Blanket suggests GenBank accession numbers, and scale bar represents the number of amino acid substitutions per site between sequences. This analysis involved 40 and 25 amino acid sequences, and a total of 575 and 541 positions in the final dataset (**(A, B)**, respectively).

Supplementary Figure 2 Subcellular fractionation of BsCAβ1:GFP and BsCAβ2:GFP in protein extracts from leaf tissues of *N. benthamiana*. Total protein extracts from leaves of *N. benthamiana* infiltrated with the indicated constructs that had been treated with 1% Triton X-100 were separated into soluble and membrane fractions by ultracentrifugation. As a control, protein extracts were separated without Triton X-100 treatment (normal condition). These fractions were analyzed by western blotting using anti-GFP, anti-VSR and anti-AALP antibodies. As controls, VSR and AALP were used as representatives of membrane and soluble proteins, respectively. T, total fraction; S, soluble fraction; P, pellet fraction.

## References

[B1] Aggarwal M. Chua T. K. Pinard M. A. Szebenyi D. M. McKenna R. (2015). Carbon dioxide “Trapped” in a β-carbonic anhydrase. Biochemistry. 54, 6631–6638. doi: 10.1021/acs.biochem.5b00987 26457866 PMC4745652

[B2] Ahn G. Kim H. Kim D. H. Hanh H. Yoon Y. Singaram I.. (2017). SH3 domain-containing protein 2 plays a crucial role at the step of membrane tubulation during cell plate formation. Plant Cell 29, 1388–1405. doi: 10.1105/tpc.17.00108 28584166 PMC5502459

[B3] Akhani H. Barroca J. Koteeva N. Voznesenskaya E. Franceschi V. Edwards G. (2005). Bienertia sinuspersici (Chenopodiaceae): a new species from Southwest Asia and discovery of a third terrestrial C4 plant without Kranz Anatomy. Systematic Bot. 30, 290–301. doi: 10.1600/0363644054223684

[B4] Altschul S. F. Gish W. Miller W. Myers E. W. Lipman D. J. (1990). Basic local alignment search tool. J. Mol. Biol. 215, 403–410. doi: 10.1016/S0022-2836(05)80360-2 2231712

[B5] Atkins C. A. (1974). Occurrence and some properties of carbonic anhydrases from legume root nodules. Phytochem 13, 93–98. doi: 10.1016/S0031-9422(00)91273-1

[B6] Atkins C. A. Patterson B. D. Graham D. (1972). Plant carbonic anhydrases, II. Preparation and some properties of monocotyledon and dicotyledon enzyme types. Plant Physiol. 50, 218–223. doi: 10.1104/pp.50.2.218 16658145 PMC366113

[B7] Badger M. R. Kaplan A. Berry J. A. (1980). Internal inorganic carbon pool of Chlamydomonas reinhardtii: evidence for a carbon dioxide-concentrating mechanism. Plant Physiol. 66, 407–413. doi: 10.1104/pp.66.3.407 16661446 PMC440644

[B8] Batistic O. (2012). Genomics and localization of the Arabidopsis DHHC-cysteine-rich domain S-acyltransferase protein family. Plant Physiol. 160, 1597–1612. doi: 10.1104/pp.112.203968 22968831 PMC3490592

[B9] Blaskovic S. Blanc M. van der Goot F. G. (2013). What does S-palmitoylation do to membrane proteins? FEBS J. 280, 2766–2774. doi: 10.1111/febs.12263 23551889

[B10] Conibear E. Davis N. G. (2010). Palmitoylation and depalmitoylation dynamics at a glance. J. Cell Sci. 123, 4007–4010. doi: 10.1242/jcs.059287 21084560 PMC2987437

[B11] Dascher C. Balch W. E. (1994). Dominant inhibitory mutants of ARF1 block endoplasmic reticulum to Golgi transport and trigger disassembly of the Golgi apparatus. J. Biol. Chem. 269, 1437–1448. doi: 10.1016/S0021-9258(17)42277-0 8288610

[B12] DiMario R. J. Clayton H. Mukherjee A. Ludwig M. Moroney J. V. (2017). Plant carbonic anhydrases: structures, locations, evolution, and physiological roles. Mol. Plant 10, 30–46. doi: 10.1016/j.molp.2016.09.001 27646307 PMC5226100

[B13] DiMario R. J. Quebedeaux J. C. Longstreth D. J. Dassanayake M. Hartman M. M. Moroney J. V. (2016). The cytoplasmic carbonic anhydrases βCA2 and βCA4 are required for optimal plant growth at low CO_2_ . Plant Physiol. 171, 280–293. doi: 10.1104/pp.15.01990 26993617 PMC4854698

[B14] Edgar R. C. (2004). MUSCLE: multiple sequence alignment with high accuracy and high throughput. Nucleic Acids Res. 32, 1792–1797. doi: 10.1093/nar/gkh340 15034147 PMC390337

[B15] Edwards G. E. Franceschi V. R. Voznesenskaya E. V. (2004). Single-cell C4 photosynthesis versus the dual-cell (Kranz) paradigm. Annl Rev. Plant Biol. 55, 173–196. doi: 10.1146/annurev.arplant.55.031903.141725 15377218

[B16] Eubel H. Braun H. P. Millar A. H. (2005). Blue-native PAGE in plants: a tool in analysis of protein-protein interactions. Plant Methods 1, 11. doi: 10.1186/1746-4811-1-11 16287510 PMC1308860

[B17] Fabre N. Reiter I. M. Becuwe-Linka N. Genty B. Rumeau D. (2007). Characterization and expression analysis of genes encoding alpha and beta carbonic anhydrases in Arabidopsis. Plant Cell Environ. 30, 617–629. doi: 10.1111/j.1365-3040.2007.01651.x 17407539

[B18] Fouracre J. P. Ando S. Langdale J. A. (2014). Cracking the Kranz enigma with systems biology. J. Exp. Bot. 65, 3327–3339. doi: 10.1093/jxb/eru015 24510938

[B19] Furbank R. T. (2017). Walking the C4 pathway: past, present, and future. J. Exp. Bot. 68, 4057–4066. doi: 10.1093/jxb/erx006 28110279

[B20] Giordano M. Beardall J. Raven J. A. (2005). CO_2_ concentrating mechanisms in algae: mechanisms, environmental modulation, and evolution. Annu. Rev. Plant Biol. 56, 99–131. doi: 10.1146/annurev.arplant.56.032604.144052 15862091

[B21] Grabherr M. G. Haas B. J. Yassour M. Levin J. Z. Thompson D. A. Amit I.. (2011). Trinity: reconstructing a full-length transcriptome without a genome from RNA-Seq data. Nat. Biotechnol. 29, 644–652. doi: 10.1038/nbt.1883 21572440 PMC3571712

[B22] Gutierrez M. Huber S. C. Ku S. B. Kanai R. Edwards G. E. (1974). Intracellular localization of carbon metabolism in mesophyll cells of C_4_ plants. Proc. Third Int. Congress Photosynthesis, 1219–1230.

[B23] Hatch M. D. Burnell J. N. (1990). Carbonic anhydrase activity in leaves and its role in the first step of C(4) photosynthesis. Plant Physiol. 93, 825–828. doi: 10.1104/pp.93.2.825 16667544 PMC1062591

[B24] Häusler R. E. Hirsch H. J. Kreuzaler F. Peterhänsel C. (2002). Overexpression of C(4)-cycle enzymes in transgenic C(3) plants: a biotechnological approach to improve C(3)-photosynthesis. J. Exp. Bot. 53, 591–607. doi: 10.1093/jexbot/53.369.591 11886879

[B25] Hemsley P. A. (2017). An outlook on protein S-acylation in plants: what are the next steps? J. Exp. Bot. 68, 3155–3164. doi: 10.1093/jxb/erw497 28158736

[B26] Hemsley P. A. Grierson C. S. (2008). Multiple roles for protein palmitoylation in plants. Trends Plant Sci. 13, 295–302. doi: 10.1016/j.tplants.2008.04.006 18501662

[B27] Hewett-Emmett D. Tashian R. E. (1996). Functional diversity, conservation, and convergence in the evolution of the alpha-, beta-, and gamma-carbonic anhydrase gene families. Mol. Phylogenet Evol. 5, 50–77. doi: 10.1006/mpev.1996.0006 8673298

[B28] Hirao T. Watanabe A. Kurita M. Kondo T. Takata K. (2009). A frameshift mutation of the chloroplast matK coding region is associated with chlorophyll deficiency in the Cryptomeria japonica virescent mutant Wogon-Sugi. Curr. Genet. 55, 311–321. doi: 10.1007/s00294-009-0247-9 19449186 PMC2691868

[B29] Hu H. Boisson-Dernier A. Israelsson-Nordstrom M. Bohmer M. Xue S. Ries A.. (2010). Carbonic anhydrases are upstream regulators of CO_2_-controlled stomatal movements in guard cells. Nat. Cell Biol. 12, 87–93. doi: 10.1038/ncb2009 20010812 PMC2906259

[B30] Hurst C. H. Hemsley P. A. (2015). Current perspective on protein S-acylation in plants: more than just a fatty anchor? J. Exp. Bot. 66, 1599–1606. doi: 10.1093/jxb/erv053 25725093

[B31] Jin J. B. Kim Y. A. Kim S. J. Lee S. H. Kim D. H. Cheong G. W.. (2001). A new dynamin-like protein, ADL6, is involved in trafficking from the trans-Golgi network to the central vacuole in Arabidopsis. Plant Cell 13, 1511–1526. doi: 10.1105/TPC.000534 11449048 PMC139540

[B32] Kando D. Ruhil K. Govindjee G. Tripathy B. C. (2022). Overexpression of cytoplasmic C4 Flaveria bidentis carbonic anhydrase in C3 Arabidopsis thaliana increases amino acids, photosynthetic potential, and biomass. Plant Biotechnol. J. 8, 1518–1532. doi: 10.1111/pbi.13830 PMC9342616 35467074

[B33] Kang H. Kim S. Y. Song K. Sohn E. J. Lee Y. Lee D. W.. (2012). Trafficking of vacuolar proteins: the crucial role of Arabidopsis vacuolar protein sorting 29 in recycling vacuolar sorting receptor. Plant Cell 24, 5058–5073. doi: 10.1105/tpc.112.103481 23263768 PMC3556975

[B34] Kant S. Seneweera S. Rodin J. Materne M. Burch D. Rothstein S. J.. (2012). Improving yield potential in crops under elevated CO(2): Integrating the photosynthetic and nitrogen utilization efficiencies. Front. Plant Sci. 3, 162. doi: 10.3389/fpls.2012.00162 22833749 PMC3400048

[B35] Kim D. H. Eu Y.-J. Yoo C. M. Kim Y.-W. Pih K. T. Jin J. B.. (2001). Trafficking of Phosphatidylinositol 3-Phosphate from the trans-Golgi Network to the Lumen of the Central Vacuole in Plant Cells. The Plant Cell. 13, 287–3001. doi: 10.1105/tpc.13.2.287 11226186 PMC102243

[B36] Kimber M. S. Pai E. F. (2000). The active site architecture of Pisum sativum beta-carbonic anhydrase is a mirror image of that of alpha-carbonic anhydrases. EMBO J. 19, 1407–1418. doi: 10.1093/emboj/19.7.1407 10747009 PMC310211

[B37] Koteyeva N. K. Voznesenskaya E. V. Berry J. O. Cousins A. B. Edwards G. E. (2016). The unique structural and biochemical development of single cell C4 photosynthesis along longitudinal leaf gradients in Bienertia sinuspersici and Suaeda aralocaspica (Chenopodiaceae). J. Exp. Bot. 67, 2587–2601. doi: 10.1093/jxb/erw082 26957565 PMC4861011

[B38] Kumar S. Stecher G. Li M. Knyaz C. Tamura K. (2018). MEGA X: molecular evolutionary genetics analysis across computing platforms. Mol. Biol. Evol. 35, 1547–1549. doi: 10.1093/molbev/msy096 29722887 PMC5967553

[B39] Kwon Y. Shen J. Lee M. H. Geem K. G. Jiang L. Hwang I. (2018). AtCAP2 is crucial for lytic vacuole biogenesis during germination by regulating vacuolar protein trafficking. Proc. Natl. Acad. Sci. 115, E1675–E1683. doi: 10.1073/pnas.1717204115 29378957 PMC5816184

[B40] Lara M. V. Offermann S. Smith M. Okita T. W. C. Andreo S. Edwards G. E. (2008). Leaf development in the single-cell C4 system in Bienertia sinuspersici: expression of genes and peptide levels for C4 metabolism in relation to chlorenchyma structure under different light conditions. Plant Physiol. 148, 593–610. doi: 10.1104/pp.108.124008 18667722 PMC2528127

[B41] Lee H. K. Cho S. K. Son O. Xu Z. Hwang I. Kim W. T. (2009). Drought stress-induced Rma1H1, a RING membrane-anchor E3 ubiquitin ligase homolog, regulates aquaporin levels via ubiquitination in transgenic Arabidopsis plants. Plant Cell 21, 622–641. doi: 10.1105/tpc.108.061994 19234086 PMC2660634

[B42] Lee D. W. Kim J. K. Lee S. Choi S. Kim S. Hwang I. N. (2008). Arabidopsis nuclear-encoded plastid transit peptides contain multiple sequence subgroups with distinctive chloroplast-targeting sequence motifs. Plant Cell 20, 1603–1622. doi: 10.1105/tpc.108.060541 18552198 PMC2483360

[B43] Lee D. W. Lee S. Lee G. J. Lee K. H. Kim S. Cheong G. W.. (2006). Functional characterization of sequence motifs in the transit peptide of Arabidopsis small subunit of rubisco. Plant Physiol. 140, 466–483. doi: 10.1104/pp.105.074575 16384899 PMC1361317

[B44] Lee M. H. Min M. K. Lee Y. J. Jin J. B. Shin D. H. Kim D. H.. (2002). ADP-ribosylation factor 1 of Arabidopsis plays a critical role in intracellular trafficking and maintenance of endoplasmic reticulum morphology in Arabidopsis. Plant Physiol. 129, 1507–1520. doi: 10.1104/pp.003624 12177464 PMC166739

[B45] Lee M. H. Yoo Y. J. Kim D. H. Hanh N. H. Kwon Y. Hwang I. (2017). The prenylated rab GTPase receptor PRA1.F4 contributes to protein exit from the golgi apparatus. Plant Physiol. 174, 1576–1594. doi: 10.1104/pp.17.00466 28487479 PMC5490915

[B46] Lee D. W. Yoo Y. J. Razzak M. A. Hwang I. (2018). Prolines in transit peptides are crucial for efficient preprotein translocation into chloroplasts. Plant Physiol. 176, 663–677. doi: 10.1104/pp.17.01553 29158328 PMC5761803

[B47] Leegood R. C. (2002). C4 photosynthesis: principles of CO_2_ concentration and prospects for its introduction into C3 plants. J. Exp. Bot. 53, 581–590. doi: 10.1093/jexbot/53.369.581 11886878

[B48] Li Y. Scott R. Doughty J. Grant M. Qi B. (2016). Protein S-acyltransferase 14: a specific role for palmitoylation in leaf senescence in Arabidopsis. Plant Physiol. 170, 415–128. doi: 10.1104/pp.15.00448 26537563 PMC4704564

[B49] Lung S. C. Yanagisawa M. Chuong S. D. (2011). Protoplast isolation and transient gene expression in the single-cell C4 species, Bienertia sinuspersici. Plant Cell Rep. 30, 473–484. doi: 10.1007/s00299-010-0953-2 21103876

[B50] McGinn P. J. Morel F. M. M. (2008). Expression and regulation of carbonic anhydrases in the marine diatom Thalassiosira pseudonana and in natural phytoplankton assemblages from Great Bay, New Jersey. Physiologia Plantarum 133, 78–91. doi: 10.1111/j.1399-3054.2007.01039.x 18405334

[B51] Medrano H. Escalona J. M. Bota J. Gulías J. Flexas J. (2002). Regulation of photosynthesis of C3 plants in response to progressive drought: stomatal conductance as a reference parameter. Ann. Bot. 89, 895–905. doi: 10.1093/aob/mcf079 12102515 PMC4233802

[B52] Moroney J. V. Bartlett S. G. Samuelsson G. (2001). Carbonic anhydrases in plants and algae. Plant Cell Environ. 24, 141–153. doi: 10.1111/j.1365-3040.2001.00669.x

[B53] Moroney J. V. Husic H. D. Tolbert N. E. Kitayama M. Manuel L. J. Togasaki R. K. (1989). Isolation and characterization of a mutant of Chlamydomonas reinhardtii deficient in the CO_2_ concentrating mechanism. Plant Physiol. 89, 897–903. doi: 10.1104/pp.89.3.897 16666639 PMC1055941

[B54] Nadolski M. J. Linder M. E. (2007). Protein lipidation. FEBS J. 274, 5202–5210. doi: 10.1111/j.1742-4658.2007.06056.x 17892486

[B55] Nelson B. K. Cai X. Nebenfuhr A. (2007). A multicolored set of *in vivo* organelle markers for co-localization studies in Arabidopsis and other plants. Plant J. 51, 1126–1136. doi: 10.1111/j.1365-313X.2007.03212.x 17666025

[B56] Offermann S. Friso G. Doroshenk K. A. Sun Q. Sharpe R. M. Okita T. W.. (2015). Developmental and subcellular organization of single-Cell C(4) photosynthesis in Bienertia sinuspersici determined by large-scale proteomics and cDNA assembly from 454 DNA sequencing. J. Proteome Res. 14, 2090–2108. doi: 10.1021/pr5011907 25772754

[B57] Offermann S. Okita T. W. Edwards G. E. (2011). Resolving the compartmentation and function of C4 photosynthesis in the single-cell C4 species Bienertia sinuspersici. Plant Physiol. 155, 1612–1628. doi: 10.1104/pp.110.170381 21263039 PMC3091117

[B58] Ohno Y. Kihara A. Sano T. Igarashi Y. (2006). Intracellular localization and tissue-specific distribution of human and yeast DHHC cysteine-rich domain-containing proteins. Biochim. Biophys. Acta 1761, 474–483. doi: 10.1016/j.bbalip.2006.03.010 16647879

[B59] Park H. Song B. Morel F. M. M. (2007). Diversity of the cadmium-containing carbonic anhydrase in marine diatoms and natural waters. Environ. Microbiol. 9, 403–413. doi: 10.1111/j.1462-2920.2006.01151.x 17222138

[B60] Park Y. Xu Z. Y. Kim S. Y. Lee J. Choi B. Lee J.. (2016). Spatial regulation of ABCG25, an ABA exporter, is an important component of the mechanism controlling cellular ABA levels. Plant Cell 28, 2528–2544. doi: 10.1105/tpc.16.00359 27697789 PMC5134978

[B61] Parry M. A. Andralojc P. J. Scales J. C. Salvucci M. E. Carmo-Silva A. E. Alonso H.. (2013). Rubisco activity and regulation as targets for crop improvement. J. Exp. Bot. 64, 717–730. doi: 10.1093/jxb/ers336 23162118

[B62] Pego J. V. Kortstee A. J. Huijser C. Smeekens S. C. (2000). Photosynthesis, sugars and the regulation of gene expression. J. Exp. Bot. 51, 407–416. doi: 10.1093/jexbot/51.suppl_1.407 10938849

[B63] Rawsthorne S. (1992). Towards an understanding of C3-C4 photosynthesis. Essays Biochem. 27, 135–146. 1425599

[B64] Razzak M. A. Lee J. Lee D. W. Kim J. H. Yoon H. S. Hwang I. (2019). Expression of seven carbonic anhydrases in red alga Gracilariopsis chorda and their subcellular localization in a heterologous system, Arabidopsis thaliana. Plant Cell Rep. 38, 147–159. doi: 10.1007/s00299-018-2356-8 30446790

[B65] Reisinger V. Eichacker L. A. (2008). Solubilization of membrane protein complexes for blue native PAGE. J. Proteomics 71, 277–283. doi: 10.1016/j.jprot.2008.05.004 18573355

[B66] Rocks O. Gerauer M. Vartak N. Koch S. Huang Z. P. Pechlivanis M.. (2010). The palmitoylation machinery is a spatially organizing system for peripheral membrane proteins. Cell 141, 458–471. doi: 10.1016/j.cell.2010.04.007 20416930

[B67] Rowlett R. S. (2010). Structure and catalytic mechanism of the beta-carbonic anhydrases. Biochim. Biophys. Acta 1804, 362–373. doi: 10.1016/j.bbapap.2009.08.002 19679201

[B68] Sohn E. J. Kim E. S. Zhao M. Kim S. J. Kim H. Kim Y.-W.. (2003). Rha1, an Arabidopsis Rab5 homolog, plays a critical role in the vacuolar trafficking of soluble cargo proteins. Plant Cell 15, 1057–1070. doi: 10.1105/tpc.009779 12724533 PMC153716

[B69] Voznesenskaya E. V. Franceschi V. R. Kiirats O. Artyusheva E. G. Freitag H. Edwards G. E. (2002). Proof of C4 photosynthesis without Kranz anatomy in Bienertia cycloptera (Chenopodiaceae). Plant J. 31, 649–662. doi: 10.1046/j.1365-313X.2002.01385.x 12207654

[B70] Voznesenskaya E. V. Franceschi V. R. Kiirats O. Freitag H. Edwards G. E. (2001). Kranz anatomy is not essential for terrestrial C4 plant photosynthesis. Nature 414, 543. doi: 10.1038/35107073 11734854

[B71] Wimmer D. Bohnhorst P. Impe D. Hwang I. Offermann S. (2019). Agrobacteria mediated transient transformation of Bienertia sinuspersici to assay recombinant protein distribution between dimorphic chloroplasts. Plant Cell Rep. 38, 779–782. doi: 10.1007/s00299-019-02375-4 30661085

[B72] Wimmer D. Bohnhorst P. Shekhar V. Hwang I. Offermann S. (2017). Transit peptide elements mediate selective protein targeting to two different types of chloroplasts in the single-cell C4 species Bienertia sinuspersici. Sci. Rep. 7, 41187. doi: 10.1038/srep41187 28112241 PMC5253730

[B73] Yoo Y. J. Lee H. K. Han W. Kim D. H. Lee M. Jeon J.. (2016). Interactions between transmembrane helices within monomers of the aquaporin AtPIP2;1 play a crucial role in tetramer formation. Mol. Plant 6, 1004–1017. doi: 10.1016/j.molp.2016.04.012 27142778

[B74] Zhou F. Xue Y. Yao X. Xu Y. (2006). CSS-Palm: palmitoylation site prediction with a clustering and scoring strategy (CSS). Bioinformatics 22, 894–896. doi: 10.1093/bioinformatics/btl013 16434441

[B75] Zuckerkandl E. Pauling L. (1965). Evolutionary divergence and convergence in proteins. Eds. Bryson V. Vogel H. J. (New York: Academic Press), 97–166.

