# Supplementary figures and images for: Expression and localization of two β-carbonic anhydrases in Bienertia, a single-cell C4 plant
Source: Front Plant Sci. 2025 Jan 16;15:1506375. doi: 10.3389/fpls.2024.1506375 (PMC11779723; doi:10.3389/fpls.2024.1506375)

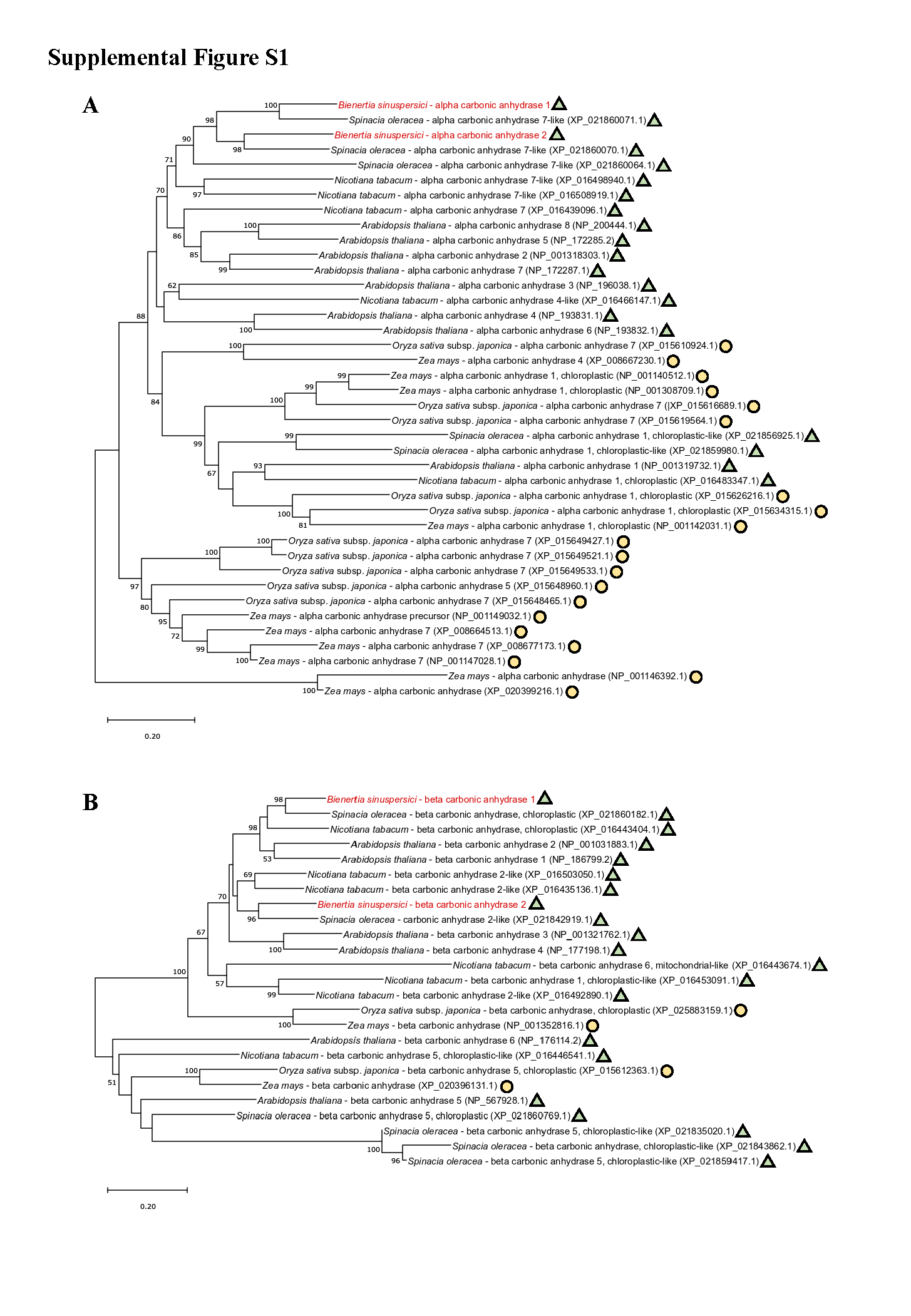

Supplement: Supplementary Figure 1 — Phylogenetic trees of carbonic anhydrases of Bienertia. Neighbor-Joining trees of α-type carbonic anhydrases (A) and β-type carbonic anhydrases (B) for Bienertia sinuspersici (red), compared to paralogous proteins of representative monocots and eudicots. All monocots and eudicots are marked as yellow circles and green triangles, respectively. These trees were computed using the Poisson correction method with pairwise deletion, and only > 50% bootstrap support are shown within 1000 replicates. Blanket suggests GenBank accession numbers, and scale bar represents the number of amino acid substitutions per site between sequences. This analysis involved 40 and 25 amino acid sequences, and a total of 575 and 541 positions in the final dataset ((A, B), respectively). [file Image1.tif]

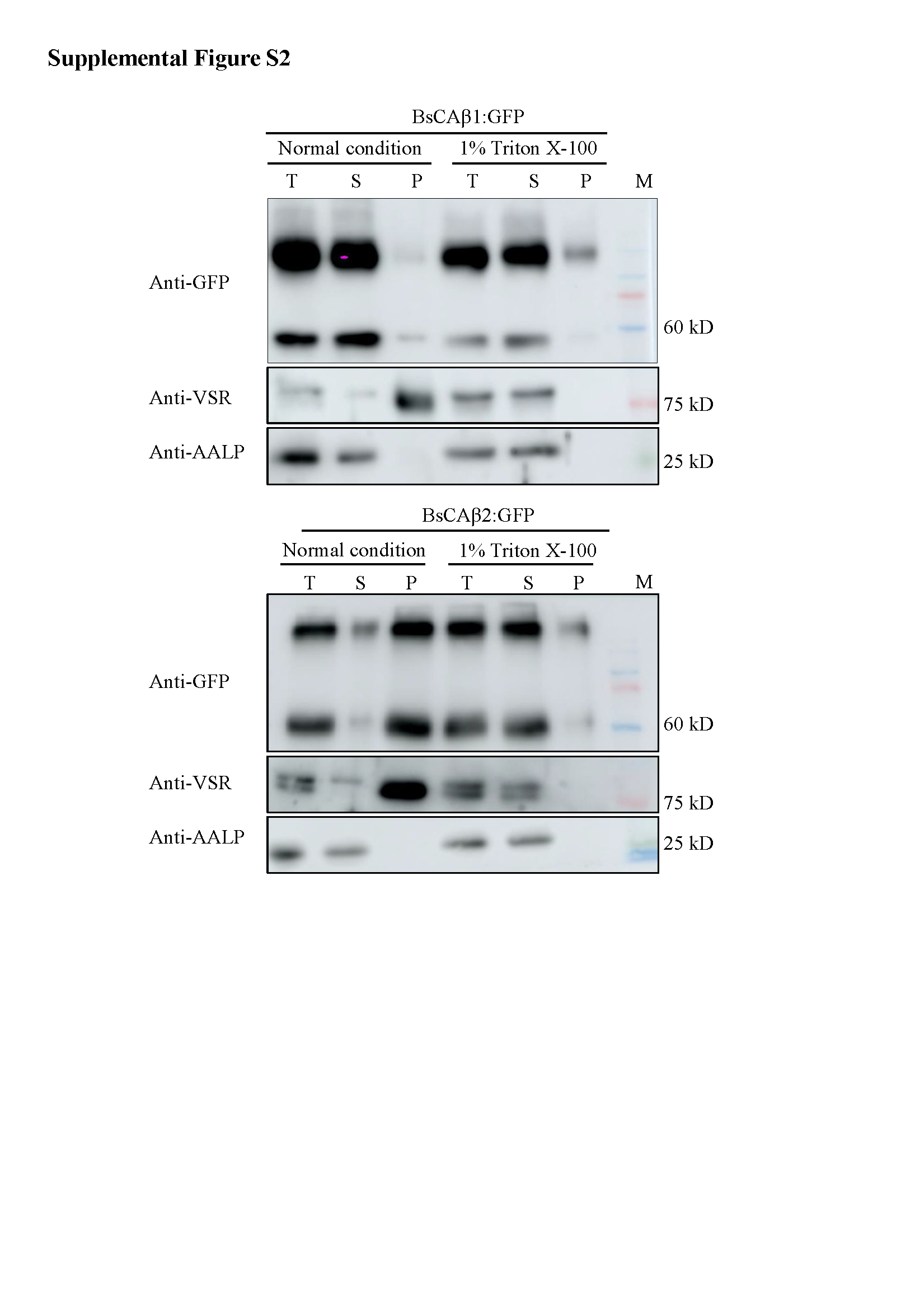

Supplement: Supplementary Figure 2 — Subcellular fractionation of BsCAβ1:GFP and BsCAβ2:GFP in protein extracts from leaf tissues of N. benthamiana. Total protein extracts from leaves of N. benthamiana infiltrated with the indicated constructs that had been treated with 1% Triton X-100 were separated into soluble and membrane fractions by ultracentrifugation. As a control, protein extracts were separated without Triton X-100 treatment (normal condition). These fractions were analyzed by western blotting using anti-GFP, anti-VSR and anti-AALP antibodies. As controls, VSR and AALP were used as representatives of membrane and soluble proteins, respectively. T, total fraction; S, soluble fraction; P, pellet fraction. [file Image2.tif]
