# Supplementary material for: Expression and localization of two β-carbonic anhydrases in Bienertia, a single-cell C4 plant
Source: Front Plant Sci. 2025 Jan 16;15:1506375. doi: 10.3389/fpls.2024.1506375 (PMC11779723; doi:10.3389/fpls.2024.1506375)
Supplement: Supplementary file 4 [file Table1.docx]

**Table S1. List of primers used in this study**

| Name | Sequence (5’ to 3’) |
| --- | --- |
| BsCAβ1-Xho1-F | CCGCTCGAGATTATTACATCAAAACAAAAAATGGCATATGAAGAAGCCATTGCC |
| BsCAβ1-Xma1-R | CCCCCCGGGCTTGATACAAGCTGAGAAGGAGAATT |
| BsCAβ2-Xho1-F | CCGCTCGAGATTATTACATCAAAACAAAAAATGGCAGGAAGCTTTAAGAAATCC |
| BsCAβ2-Xma1-R | CCCCCCGGGCTTGCTCCAATAGAAGGTGAAAAAGT |
| BsCAβ2 (C13,14S)-Xho1-F | CCGCTCGAGATGGCAGGAAGCTTTAAGAAATCCATGGCCATGATATCTTCTGCAAAACAACTTCCG |
| BsCAβ2 (C13,14S)-Xma1-R | CCCCCCGGGCTTGCTCCAATAGAAGGTGAAAAAGT |
| Arf1-Xho1-F | CCGCTCGAGATGGGGTTGTCATTCGGAAAG |
| Arf1-BamH1-R | CGGGATCCTGCCTTGCTTGCGATGTTG |
| Arf1[T31N]-F | ATGCTGCTGGTAAGAACACTATCCTCTACAAG |
| Arf1[T31N]-R | CTTGTAGAGGATAGTGTTCTTACCAGCAGCAT |
| qRT-BsCAβ1-F | TAAAGCCGGATCAAAAGCGGT |
| qRT-BsCAβ1-R | CCCTTCCTTCATTCTCTTAATCGGA |
| qRT-BsCAβ2-F | ACGTGGCAGCTGCAAAGATA |
| qRT-BsCAβ2-R | TGTCACTGGTACCGCCAATATA |
| qRT-BsPPDK-F | GGGATTGGCTTTCTCTTAATGGA |
| qRT-BsPPDK-R | AGGTGGTGAAAGCGGTTCTTT |
| qRT-BsCAα1-F | GTTCAAGTAATACCCAAAGCTGAGG |
| qRT-BsCAα1-R | GTCTTCATTCTCCCACTCAATCATG |
| qRT-BsCAα2-F | GGTCACGATATTGAGATCGTATGGA |
| qRT-BsCAα2-R | GTATGCTCTGATGGTGAGTGCC |
